# Variations in orientation relationships between rutile inclusions and garnet host relate to magmatic growth zoning

**DOI:** 10.1007/s00410-024-02146-9

**Published:** 2024-06-17

**Authors:** Victoria Kohn, Thomas A. Griffiths, Taisia Alifirova, Nina Daneu, Olga Ageeva, Rainer Abart, Gerlinde Habler

**Affiliations:** 1 Department of Lithospheric Research, University of Vienna, Josef-Holaubek-Platz 2, Vienna, 1090 Austria; 2 Jožef Stefan Institute, Jamova cesta 39, Ljubljana, 1000 Slovenia

**Keywords:** Rutile inclusions, Crystallographic orientation relationships, Shape orientation relationships, Garnet microstructural zoning

## Abstract

Rutile inclusions in almandine-spessartine garnet from a peraluminous pegmatoid from the Moldanubian zone (Bohemian Massif, AT) show distinct changes in aspect ratio, shape preferred orientations (SPO) and crystallographic orientation relationships (COR) along the transition between microstructurally different growth zones in the garnet core and rim. For identification of the COR characteristics we pool specific CORs based on their common axial relationship into three COR groups: Group 103_R_/111_G_, Group 001_R_/111_G_ and Group 001_R_/100_G_. The rutile inclusions in the garnet core domains are elongated along the four Grt$\langle$111$\rangle$ directions and are dominated by COR Group 103_R_/111_G_. The garnet rim zone additionally contains rutile needles elongated along Grt$\langle$100$\rangle$. Here, Group 001_R_/111_G_ and 001_R_/100_G_ are more abundant than in the garnet core. Needle-shaped rutile in the rim shows a systematic correlation between SPOs and CORs as needles elongated parallel to Grt$\langle$111$\rangle$ are dominated by Group 103_R_/111_G_ and 001_R_/111_G_, whereas those needles elongated parallel to Grt$\langle$100$\rangle$ exclusively pertain to CORs of 001_R_/100_G_. Furthermore, the frequency of each particular SPO in the garnet rim clearly depends on the local growth direction of the particular Grt{112} sector. Facet-specific variations in rutile SPO frequencies in different sectors and growth zones of garnet were observed even between equivalent directions, indicating that the microstructures and textures of rutile inclusions reflect varying parameters of garnet growth. The characteristic differences in COR groups of different garnet growth zones are referred to compositional changes in the bulk melt or compositional boundary layer, associated with magmatic fractional crystallisation.

**Supplementary Information:**

The online version contains supplementary material available at 10.1007/s00410-024-02146-9.

## Introduction

Mineral inclusion−host systems have great potential to record information on multiple stages of the structural and compositional evolution of rocks and associated phase relationships, which are used to decipher geological histories (e.g. Ferrero and Angel 2018 with references). In the current study, a ’mineral inclusion’ is denoted as a crystal or a crystalline aggregate of one or more phase(s) entirely enclosed by a single crystal of another phase representing the host, without any preliminary genetic implications. As inclusion phases may form before, during, or after host crystallisation, knowledge about the relative timing of formation of the involved phases is crucial in order to accurately interpret petrological data obtained from them. For example, inclusions originating from pre-existing mineral phases that were entrapped in a host crystal (overgrowth) may still reflect conditions prior to host growth (e.g. Spiess et al. 2007), or provide information on the stage of enclosure (e.g. Galwey and Jones 1962; Rice et al. 2006; Skrzypek et al. 2011) or subsequent reequilibration (e.g. Griffiths et al. 2014; Cesare et al. 2021). Inclusions that formed simultaneously with the host (co-growth, co-precipitation) may record conditions during host crystallisation (e.g. Burton 1986; Wang et al. 1999; Hopkins et al. 2008; Hwang et al. 2015; Griffiths et al. 2020) whereas inclusions originating from re-equilibration of a pre-existing host (exsolution, intragranular precipitation) may yield information on changes in the physico-chemical conditions after the host phase had formed (e.g. Hwang et al. 2007; Proyer et al. 2009; Hwang et al. 2007; Harlov 2011; Proyer et al. 2013; Axler and Ague 2015a; Ageeva et al. 2016, 2017; Hwang et al. 2019) and/or can trace the composition of the unaltered precursor of the host crystal, when the chemical components of the inclusion phases previously had been dissolved in this phase (e.g. Van Roermund et al. 2000; Dobrzhinetskaya et al. 2002; Zhang et al. 2003; Xu et al. 2015; Ackerson et al. 2017; Keller and Ague 2019, 2020). Petrogenetic footprints that allow to unequivocally determine the temporal relationship between inclusion formation and host crystallisation are required, as misinterpreted phase relationships may lead to severe geological misconceptions. Especially, elevated Ti-content in garnet serves as indicator of UHP/UHT conditions (Zhang et al. 2003; Ackerson et al. 2017), rendering knowledge of the correct timing relationships of the formation of rutile inclusions and their garnet host crucial (Keller and Ague 2019; Griffiths et al. 2020). These timing relationships are commonly inferred from compositional and microstructural characteristics, as are compositional zoning in the host phase along with the spatial distribution of inclusion phases, their size distribution, and the assemblages of polymineralic inclusions (Perchuk 2008; Griffiths et al. 2014; Axler and Ague 2015a, b). Importantly, inclusion habits, and the presence/absence of shape orientation relationships between needle-shaped rutile inclusions and their host garnet are used as markers for the identification of potential interactions between the host and inclusion lattices (Hwang et al. 2007; Ague and Eckert 2012; Axler and Ague 2015a). An increasing number of studies also involve investigation of crystallographic orientation relationships between rutile inclusions and garnet host (Guinel and Norton 2006; Hwang et al. 2007; Proyer et al. 2013; Hwang et al. 2015, 2016; Griffiths et al. 2016; Xu and Wu 2017; Hwang et al. 2019; Keller and Ague 2019; Griffiths et al. 2020; Keller and Ague 2020, 2022). Crystallographic orientation relationships (CORs) are defined as “systematic relationships between the crystallographic orientations of next-neighbour crystal pairs sharing boundary segments” (Habler and Griffiths 2017).

The existence of systematic shape orientation relationships (SORs) and/or crystallographic orientation relationships (CORs) between needle-shaped or platy inclusions and their host clearly allows to exclude inclusion formation by overgrowth, as the presence of the garnet lattice during oriented nucleation of the inclusion phase is required (Keller and Ague 2019; Griffiths et al. 2020). Observations of such lattice interactions between garnet host and rutile inclusions in the literature have been frequently interpreted as indicative of inclusion formation by precipitation within a pre-existing garnet crystal (Gou et al. 2014; Keller and Ague 2019, 2022). Proposed mechanisms of this timing relationship include exsolution (Griffin et al. 1971), open system precipitation (Proyer et al. 2013), and crystallisation from infiltrated melt/fluid along cleavages or cracks (Hwang et al. 2007, 2019), which differ by the extent of material exchange between the garnet interior and the rock matrix. Contrastingly, other studies concluded, that rutile inclusions showing SOR and COR with the garnet host may also form during growth of the host phase (Wang et al. 1999; Hwang et al. 2015; Griffiths et al. 2020). Such a co-growth origin of oriented rutile inclusions has been hypothesised to involve heterogeneous oriented nucleation of rutile at the garnet growth facet and subsequent co-growth of both phases (Griffiths et al. 2020).

Generally, the majority of oriented needle-shaped rutile inclusions in garnet, show shape preferred orientations of rutile defined by elongation parallel to Grt$\langle 111 \rangle$ directions (Zhang et al. 2003; Guinel and Norton 2006; Ague and Eckert 2012; Hwang et al. 2015; Griffiths et al. 2020; Keller and Ague 2022), whereas only subordinately elongation parallel to Grt$\langle 100 \rangle$ has been reported (Hwang et al. 2015). Contrasting with the small number of observed SORs between rutile and garnet, their CORs show much larger diversity. Eleven non-equivalent specific CORs have been identified between rutile inclusions and host garnets from different rock types that formed under a wide range of conditions (Hwang et al. 2016; Griffiths et al. 2020). This COR diversity renders rutile inclusions in garnet host crystals promising candidates for delivering petrogenetic information (Hwang et al. 2015; Griffiths et al. 2016; Hwang et al. 2016, 2019; Keller and Ague 2019; Griffiths et al. 2020; Keller and Ague 2022), especially as their relative frequencies vary considerably. Still, the petrogenetic parameters that promote or suppress the development of certain CORs have not yet been constrained. Furthermore, due to the lack of combined statistically representative SOR and COR data, the relationships between shape and crystallographic orientation relationships remained unclear. Whereas the SPO formation of rutile inclusions parallel Grt$\langle 111 \rangle$ directions in intragranular garnet domains can be explained by the atomic structure of garnet (Zhang et al. 2003; Proyer et al. 2013; Grew et al. 2013; Ackerson et al. 2017), the controlling parameters of COR formation between rutile and garnet still need to be deciphered (Griffiths et al. 2020; Keller and Ague 2022).

Griffiths et al. (2020) proposed that the frequencies of specific CORs between rutile inclusions and garnet host may deliver enlightening petrogenetic information, rather than the mere presence or absence of certain CORs. Along with a statistical treatment of COR characteristics, Griffiths et al. (2020) also proposed to use details of domain-specific SPO-frequencies in order to distinguish, whether inclusions formed by co-growth or at a stage of re-equilibration of the host crystal. Inclusion formation within intragranular host domains, where the host lattice imposes a 3D control on any nucleus, is expected to induce a different crystallographic interaction between the two lattices, than inclusion formation at a propagating growth facet of the host. These different lattice interactions may be reflected by the COR characteristics of rutile inclusions in garnet.

The current study aims to test these hypotheses stated by Griffiths et al. (2020) by combining statistical information from shape preferred orientations, crystallographic orientation relationships, and crystal habits of particular shape-oriented acicular rutile inclusions with microstructural, crystallographic and compositional information from their garnet host crystal. This approach is applied to a peculiar pegmatoid garnet sample with remarkably well defined microstructural growth zoning of garnet, which is reflected by numerous rutile inclusions of varying habit and size (Kohn et al. 2024). The comparison of the SPO and COR characteristics of the rutile inclusions in different crystallographically equivalent growth zones and growth sectors of the host garnet document (i) the mutual correlation of the SPO- and COR-characteristics of needle-shaped rutile inclusions in garnet, (ii) their relationship to the associated growth facet and the local growth direction of garnet, (iii) their ability to record growth zoning of garnet, and (iv) their potential to contribute to deciphering timing relationships between inclusion formation and host crystallisation.

## Sample Material

A c. 10 mm sized inclusion-bearing almandine-spessartine garnet crystal from a peraluminous pegmatoid from the Gföhl Unit in the Bohemian Massif (Austria) has been subject of a previous investigation, which deciphers the history of magmatic directed garnet growth and the relationships with the major phases in the rock matrix, comprising the primary magmatic assemblage plagioclase + garnet + kyanite + biotite (Kohn et al. 2024).

In this preceding study, three growth stages of garnet were deciphered. Compositional and microstructural data document garnet crystallisation at magmatic to subsolidus conditions during pegmatoid rock solidification, which occurred along a cooling and decompression path (Schantl et al. 2019) within a limited time window of pegmatoid formation and emplacement (Kohn et al. 2024, with references). Based on asymmetries in crystal morphology, peculiar inclusion zoning and compositional zoning of the sector zoned garnet, disequilibrium growth features were identified, and changing properties of the melt phase during garnet growth at rather high rates were inferred (Kohn et al. 2024).

The authors attribute the absence of rutile in the rock matrix, as well as inclusion-defined sector zoning of the garnet core (predominant rutile inclusions in {112}_Grt_ core sectors, and the predominance of phosphate inclusions in {110}_Grt_ core sectors) to the effect of compositional boundary layers (CBLs), allowing for the local accumulation of Ti and P, respectively, directly at the garnet-melt interface. Spheroidal nanoinclusions of an Si-rich phase in a garnet rim zone (R1-R2 boundary, supplementary Fig. S4.5 in Kohn et al. 2024) are regarded as melt inclusions, which serve as additional indicator of magmatic garnet growth. Building on the results by Kohn et al. (2024), the current study is focused on three microstructurally distinct garnet growth zones (core, transition zone and rim) in a euhedral {112}_Grt_ growth sector, which have formed sequentially in the first magmatic growth stage. This microstructurally prominent transition previously has been shown to be associated with systematic changes in trace element content (Na, Ti) and OH^–^-content in garnet, whereas changes in the major element composition of garnet are only subordinate (Kohn et al. 2024).

## Methods

### Electron probe microanalysis (EPMA)

Mineral compositions were analysed using a CAMECA SXFive field emission gun electron probe microanalyzer at the Department of Lithospheric Research of the University of Vienna (AT). The element distribution profile L2 (Fig. 2) was collected at 20 kV, 50 nA, with a defocused probe of c. 3 $\upmu$m diameter. The following detector crystals were used: LTAP (Na, P), TAP (Si, Al, Mg), LPET (Ca), LPET (Ti), and LLIF (Mn, Fe). Standardisation was done on augite for Na, Ca and Ti, on almandine for Si, Al, Fe, Mg, on apatite for P, and on spessartine for Mn. Detection limits are c. 142 ppm for Na, 56 ppm for P, 37 ppm for Ti. Standard deviations for profile L2 are $\text{Na}_{2}\text{O}$ 0.012, $\text{SiO}_2$ 0.113, $\text{Al}_2\text{O}_3$ 0.088, $\text{P}_2\text{O}_5$ 0.020, CaO 0.005, $\text{TiO}_2$ 0.100, MnO 0.218, FeO 0.175, MgO 0.061. The dataset of profile L2 is provided as supplementary material (S1 EPMA data). In domain R1 of section GarsE_I a Ti-distribution profile was measured over a distance of 66 $\upmu$m using a focused electron beam at 15 kV, 50 nA, step size 0.25 $\upmu$m and a dwell time of 2 s. Counts on the Ti K$\alpha$ peak of PET, LPET, LLIF were summed up to the total counts per second (Fig. 3). The selected domain was inclusion-free to a minimum of 10 $\upmu$m depth. The absence of nanoinclusions (< 100 nm grain size) has been proven by STEM-imaging in the frame of a related study involving the studied material (Kohn et al. 2024). The Ti-distribution profile intentionally crosses the tips of two rutile needles (Rt 1 and Rt 2, Fig. 3), which have a cross section dimension of c. 1 $\upmu$m as measured orthogonal to the trace of their elongation direction projected onto the plane of the thin section. The two rutile needles plunge at 35^∘^ with respect to the thin section plane.

### Electron backscatter diffraction (EBSD) analysis

In order to determine the shape orientation relationship (SOR) and the crystallographic orientation relationship (COR) between rutile inclusions and garnet host, the crystal orientations of garnet and rutile were determined by EBSD single point analyses using an FEI Quanta 3D FEG scanning electron microscope at the Department of Lithospheric Research of the University of Vienna (AT). The instrument is equipped with a Schottky type field emission gun electron source and an Ametek-EDAX Digiview 5 EBSD camera. A total of 354 rutile inclusions were measured. The garnet host was measured in domains R1 (n = 51), Transition zone (n = 10), {112}_Grt_ core (n = 6), {110}_Grt_ core (n = 1).

The maximum angle of misorientation of all measured garnet orientations with respect to their average orientation is 1.14^∘^, proving that the studied domains represent a single garnet crystal (Appendix Fig. 12). A table of the EBSD single point orientation data is provided as supplementary material (S2 EBSD data). During EBSD analysis, electron beam settings of 15kV, 4nA were applied to the sample at 14 mm working distance and 70^∘^ tilt. Kikuchi patterns were collected at $2\times 2$ binning of the EBSD camera ($696\times 520$ pixels resolution), using exposure times of 175 or 382 milliseconds for each image frame, and averaging over 5–8 image frames. A minimum of 3 reflectors and a maximum of 15–18 reflectors at a minimum peak distance of 9–12 pixels were used for indexing. Interplanar angles of identified reflectors with a tolerance of 2^∘^ were used for indexing by comparison with reference crystal structures for garnet (cubic Laue class m3m; a = 11.526 Å; TSL database) and rutile (di-tetragonal Laue class 4/mmm; a = b = 4.59 Å, c = 2.96 Å; TSL database). Each orientation solution was checked for indexing statistics, and in case of poor distinction of the first and second orientation solution, the Hough settings were adjusted until an unambiguous orientation determination was achieved. Thus, only statistically unique orientation solutions were included in the dataset. By correlating the reference frames of the EBSD crystal orientation data with those of the OM-images, we are able to link the elongation direction of each rutile inclusion to a specific crystal direction of garnet.

In order to identify crystallographic orientation relationships between inclusion and host crystals, the categorisation approach of (Griffiths et al. 2020, their Fig. A1) was followed, using the MTEX toolbox version 5.4.0 (Bachmann et al. 2010) for Matlab (version R2020a). Rutile inclusion orientations obtained by EBSD were filtered according to whether particular crystallographic directions or plane poles of rutile followed axial relationships with particular garnet directions, within a set angular threshold. Sequential filtering of the dataset for different axial relationships, always applying the filtering criteria (“rules”, as defined in the supplementary Table 3) in the same sequence, enabled us to determine for each rutile inclusion whether it followed one of the 12 crystallographic orientation relationships tested for. The rules and sequence used made it impossible for one inclusion to be assigned to multiple categories. The approach was modified by changing some threshold angles and by slightly altering the rules used to search for the specific crystallographic orientation relationships (CORs) “COR-6” Hwang et al. (2015, 2016) and COR “R3b” (Griffiths et al. 2016, 2020). Additionally, some extra filtering of inclusions assigned as uncategorised by the previous algorithm was carried out. For details of the categorisation procedure and all changes with respect to Griffiths et al. (2020), see supplementary sections “Description of categorisation method” and “Definition of COR-3^#^”. When applied to the EBSD datasets previously analysed by Griffiths et al. (2020), the updated thresholds and rules return almost identical results. The difference between original and updated rule sets never exceeds ± 1 inclusion per COR, and is usually zero. The combination of rutile-garnet axial relationships and corresponding threshold angles used to find each specific COR are given in supplementary Table 3. Inclusions that were not assigned to any specific COR are collected into the category “*uncategorised*”. A small number of inclusions in this category share one common axial relationship with reported CORs (denoted as uncategorised^+ax^ in the text and Table 2).

### Transmission electron microscopy (TEM)

Based on the EBSD orientation data, a TEM foil (Appendix Fig. 9) has been extracted from the outer core domain of the ($\overline{1}$21)_Grt_ sector, employing focused ion beam (FIB) preparation to generate a section perpendicular to the ($\overline{1}$21)_Grt_ plane. FIB preparation was performed using the same FEI Quanta 3D FEG instrument as for EBSD analysis. The Gallium-ion probe was operated at accelerating voltages of 30 kV during foil extraction and thinning, at 5 and 2 kV during final thinning and cleaning steps, and at varying ion probe currents from 65 nA to 27 pA, in order to reach a TEM foil thickness of 25 nm. Before transmission electron microscopy (TEM), the FIB foil was coated with a thin layer of amorphous carbon.

For high-resolution imaging we used a probe Cs-corrected scanning TEM (STEM; JEM-ARM CF, Jeol Ltd., Tokyo, Japan) with cold field-emission gun operated at 200 kV at the National Institute of Chemistry in Ljubljana, SI. High-angle annular dark field (HAADF) images were acquired at inner and outer semi-angles of 68 and 180 mrad, and the annular bright field (ABF) images at inner and outer angles of 11 and 22 mrad, respectively. The microscope is equipped with a JEOL Centurio 100 mm^2^ EDXS detector (JEOL, Tokyo, Japan), and a GIFQuantum ER dual-EELS system (GATAN-AMETEK, Pleasanton, USA) for chemical analyses at the nanoscale.

### Shape preferred orientation (SPO) count

The orientation of needle-shaped inclusions is inferred from the elongation direction of rutile needles in OM images that were collected using the same sample coordinate system as during EBSD data collection. In the OM images any needle elongation direction is observed as projected to the thin section plane. The traces of projected needle directions were correlated with the traces of selected garnet crystal directions as derived from EBSD analysis. The inclination of each rutile SPO direction with respect to the thin section plane is inferred from the length of the projected needles for a defined depth of focus of the OM image. In addition, the inclination angle of the rutile needles was checked by U-stage OM, tilting needles to an orthogonal orientation (either parallel or perpendicular) with respect to the viewing direction in the U-stage optical microscope.

Individual SPOs of rutile reflect shape orientation relationships (SORs) with garnet, due to needle elongation along $\langle 111 \rangle$_Grt_ (four crystallographic equivalent directions) and $\langle 100 \rangle$_Grt_ (three crystallographic equivalent directions). Particular elongation directions of rutile (namely, each SPO) is numbered as S$\langle 111 \rangle$_1–4_ and S$\langle 100 \rangle$_1–3_. The number of needle-shaped inclusions with a particular SPO have been counted in selected areas of OM images taken with transmitted light (TLp) applying equal depth of focus for all images. Areas were selected to be representative for the microstructure of the individual domain (transition zone and R1 zone), avoiding domains with microstructural signs of secondary overprinting and individual inclusions with tabular or equidimensional habit in the defined areas. One area in the transition zone (OM-10, $300 \times 90 \upmu$m) of growth facet ($\overline{1}$21)_Grt_, seven areas of R1 in the same sector (OM-01 to OM-07, $200 \times 300 \upmu$m each) and one area of a brownish coloured domain within R1 of the same sector (OM-08, $120 \times 190 \upmu$m) were analysed. For comparison, one area of R1 in a different garnet growth sector, corresponding to the crystallographically equivalent growth facet ($\overline{2}$11)_Grt_, has been investigated as well (OM-09, $200 \times 300 \upmu$m). The dataset of the SPO quantification is provided as supplementary material (S3 SPO data).

As rutile needles have different inclination angles with respect to the thin section surface (S$\langle 111 \rangle$_1_ = 56^∘^, S$\langle 111 \rangle$_2_ = 35^∘^, S$\langle 111 \rangle$_3_ = 31^∘^, S$\langle 111 \rangle$_4_ = 15^∘^), the effect of sample sectioning has to be considered when quantifying SPO frequencies from 2D observation. The relative frequencies of rutile needles with particular SPO were quantified as fractions of all rutile needles observed in a given area and a certain limited depth of focus. To determine the potential bias of the sectioning effect on SPO frequencies, we estimate the theoretical relative frequencies of needles with elongation direction parallel to the $\langle 111 \rangle$_Grt_ directions of the host (at the measured garnet crystal orientation in EBSD sample reference frame) assuming an identical and homogeneous equal spacing between parallel neighbouring needles for each of the observed SPOs. Assuming unit spacing of parallel adjacent needles measured orthogonal to the needle elongation in a plane that is orthogonal to the thin section plane and contains the needle elongation direction, the spacing in the thin section plane is calculated as ‘*d*’ = 1/sine θ (where ‘θ’ is the inclination angle of a needle with respect to the thin section surface). Using the inverse of ‘*d*’ as the number of inclusions intersecting the sample surface at unit spacing, the ratios of rutile needles for each of the SPO directions is derived. In the following we refer to *observed frequency* as the fraction of the counted number of inclusions observed in OM-TLp in a certain sample area and within a certain very limited depth of focus. The *estimated frequencies at unit spacing* are calculated assuming homogeneous equal distribution of inclusions considering the sectioning effect for the given host crystal orientation.

## Results

### Microstructural and compositional zoning of garnet

**Fig. 1:**
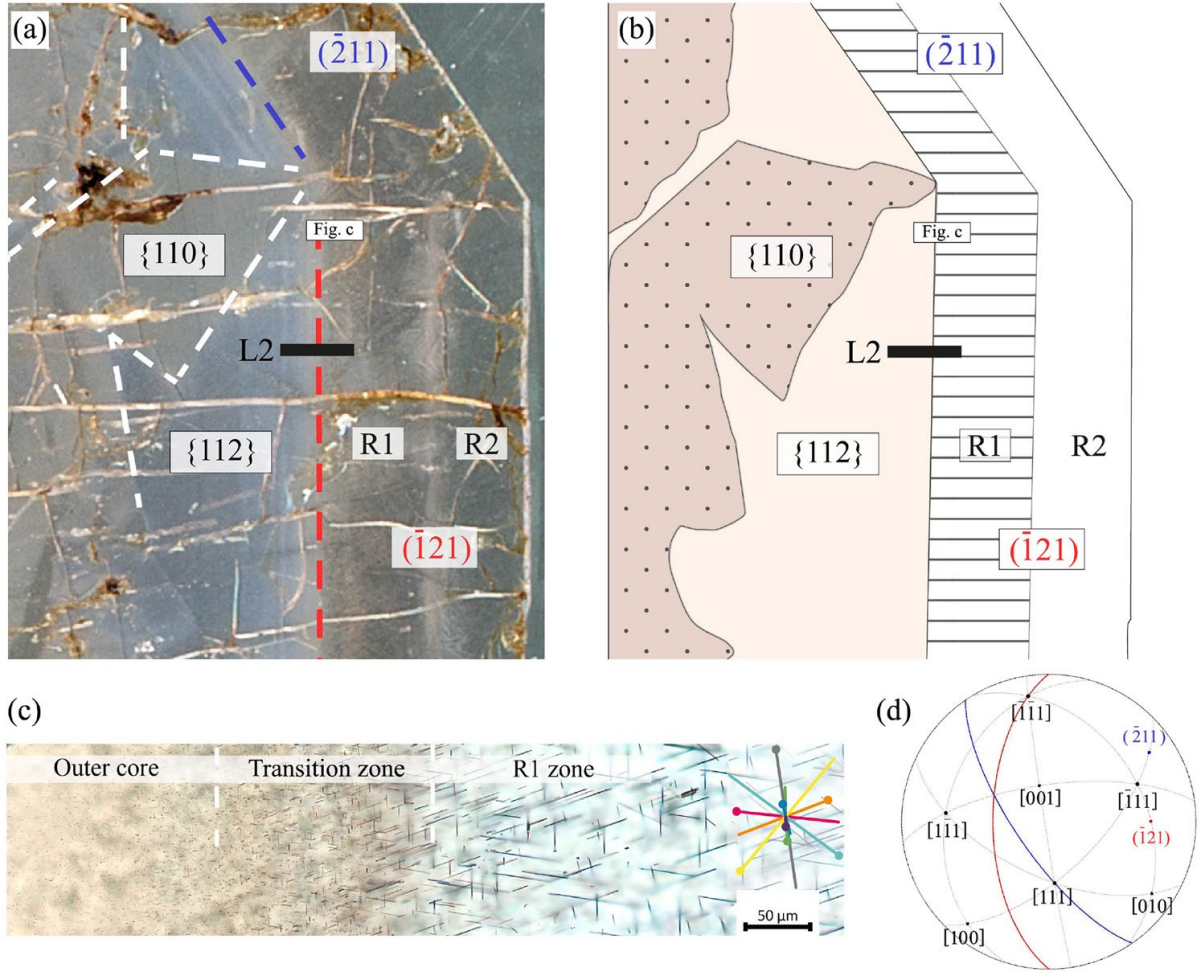
**a** Thin section scan (crossed polarized light) and **b** sketch of the studied garnet domains, representing a detail of the garnet crystal studied in its entirety by Kohn et al. (2024). The coloured garnet core consists of {112}_Grt_ and {110}_Grt_ growth sectors. Rim zones R1 and R2 are uncoloured and contain rutile needles with a high aspect ratio. R1 was investigated in two crystallographically equivalent garnet sectors ($\overline{1}$21)_Grt_ (highlighted red) and ($\overline{2}$11)_Grt_ (highlighted blue). **c** Microstructural domains along the transition from the outer garnet core (left) to R1 (right) of the ($\overline{1}$21)_Grt_ sector (position indicated in (a)) show a gradual increase in aspect ratio of the inclusions, and a decrease in their frequency. R1 contains rutile needles with shape preferred orientations (SPOs, inset on the right). Each coloured line shows a rutile needle SPO direction projected onto the image plane. Dots at the tip of each line indicate the intersection with the thin section plane of each SPO. **d** The inset pole figure (upper hemisphere, equal angle projection) shows the crystal orientation of garnet and the two relevant facets using the same reference frame as in figures **a**–**c**

The coloured garnet core consists of {112}_Grt_ growth sectors in which equant rutile inclusions are predominant, and {110}_Grt_ growth sectors in which phosphate inclusions prevail. Rim domains R1 and R2 are uncoloured, contain rutile needles with a high aspect ratio and exclusively consist of {112}_Grt_ growth sectors. The microstructure of the entire garnet crystal has been described in detail by Kohn et al. (2024), who also deciphered the magmatic growth evolution of this garnet. Based on these results, the current study sets focus on the gradual microstructural transition from the outer garnet core to the rim domain R1 ( Fig. 1), which are two subsequent microstructurally distinct growth zones of garnet in {112}_Grt_ growth sectors. From the outer core to R1 the internal microstructure of garnet successively changes over a distance of c. 100–150 $\upmu$m measured in the thin section plane, showing a diminution of garnet colouring and a change in rutile morphology (Fig. 1c). Considering the c. $30^\circ$ inclination of the ($\overline{1}21$)_Grt_ facet plane with respect to the thin section normal direction, the transition zone has a true width of c. 85–130 $\upmu$m. As the thickness of the thin section is equal or less than 60 $\upmu$m, the gradual change cannot be explained by the oblique section.

The microstructural domains shown in Fig. 1c, are considered as consecutive growth zones at the transition from the garnet core to R1 within {112}_Grt_ sectors:

(i) The outer core is constituted by brownish coloured garnet which comprises mostly equant rutile inclusions of c. 80–200 nm size (minimum cross section width). Although the rutile crystals appear equant in optical microscopy (OM), they are elongated in the order of 100s of nm when observed at higher spatial resolution in transmission electron microscopy (section ‘Observed SPO frequencies and rutile-garnet SOR’ and Appendix Fig. 9).
(ii) The transition zone between the garnet core and R1 shows gradually diminishing brownish colouring of garnet, and gradually increasing aspect ratio of the rutile inclusions towards R1. Rutile inclusions in the transition zone have minimum cross section widths of 60–250 nm, and rod-shaped inclusions with lengths in the order of a few 10s of $\upmu$m, increasing towards the R1 zone. Rutile crystals show predominantly oblique extinction (extinction at an angle with respect to the elongation direction of the rutile needle) when observed by crossed polarized transmitted light microscopy (OM-TLx).
(iii) The R1 zone of garnet is mostly colourless and contains needle-shaped rutile with a high aspect ratio, having up to 150 $\upmu$m length and about 200 nm width. In OM-TLx, straight extinction (parallel to needle elongation) is more common than in the transition zone, but oblique extinction is observed as well.

**Fig. 2:**
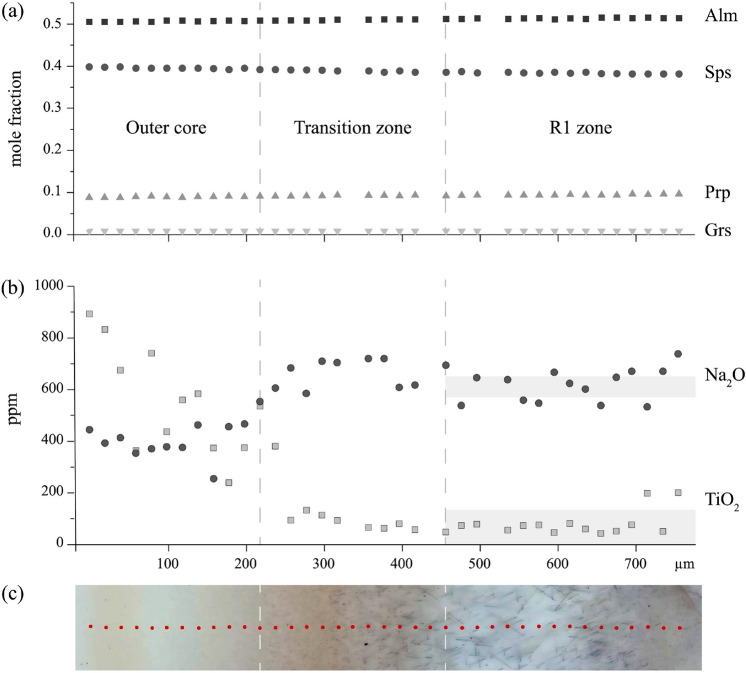
EPMA element distribution profile L2 of garnet showing details of compositional zoning along the transition from outer core to R1 (39 point measurements on a profile length of 757 $\upmu$m as marked in Fig. **c**. **a** The major element composition (given as mole fraction of endmember components Alm, Sps, Prp, Grs) is rather constant across the three microstructurally distinct zones. **b** The TiO_2_ content decreases in the outer core towards the transition zone. Na_2_O increases at the boundary between the outer core and the transition zone. Single point electron probe microanalysis (EPMA) in inclusion-free garnet in R1 yields 68–134 ppm TiO_2_ and 569–644 ppm for Na_2_O, as indicated by the grey shaded bars. **c** Photomicrograph (TLp) showing the position of the measurement spots shown in Figs. **a** and **b**

The major element composition in a detailed EPMA profile from the outer garnet core to the R1 zone, shows very subtle Fe- and Mn zoning at constantly low Mg and Ca-contents (Fig. 2a). A compositional profile across the entire garnet crystal is provided in Fig. 7 in Kohn et al. (2024). Contrasting with major element zoning, the Ti and Na contents in garnet change more pronouncedly. The Ti-content gradually decreases from > 800 ppm in the outer garnet core domain to < 200 ppm in the majority of the transition zone. The Na-content in garnet increases more abruptly at the boundary between the outer core and the transition zone from c. 400 to c. 700 ppm (Fig. 2b). Kohn et al. (2024) report an increase in the OH^–^-content in garnet from about 8 ppm in the core, to about 11–13 ppm in R1. Apparently, the microstructural change from the outer core to the R1 zone is spatially associated with systematic changes in the trace element content of garnet, whereas the major element composition of garnet is rather constant.

**Fig. 3:**
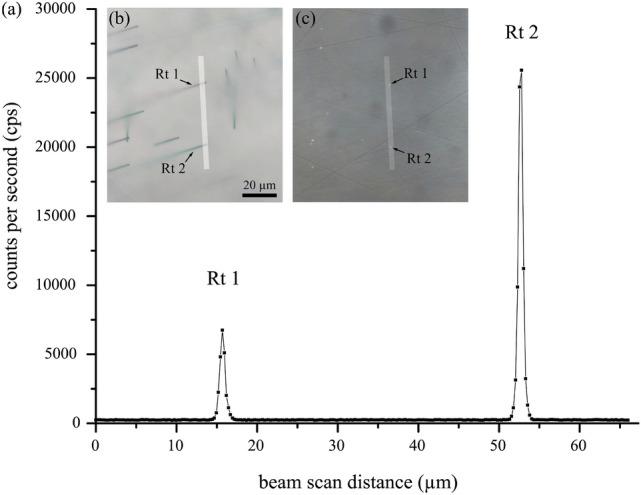
**a** EPMA line scan (derived from probe scanning) for Ti in counts per second, and the position of this 66 $\upmu$m long X-ray line scan (transparent white bar) crossing the transect of two rutile needles (Rt 1 and Rt 2) with the thin section surface in OM using **b** transmitted light and **c** reflected light

A precise EPMA X-ray line scan was performed in the R1 zone to obtain the Ti-distribution in garnet around the tips of rutile inclusions that intersect the thin section surface. The probe scan intentionally crosses the tips of two rutile needles (Rt 1 and Rt 2, Fig. 3). The background concentration of Ti in inclusion-free garnet produces between 206 and 272 counts per second (cps) with a mean of 240 ± 11 cps (1 standard deviation). The peaks of Rt 1 and Rt 2 amount to 6746 cps and 25566 cps, respectively. Ti counts increase towards the rutile inclusion within a distance of about 1–2 $\upmu$m from the rutile/garnet interface. This profile length next to the inclusion is regarded as domain where the EPMA analysis of garnet is influenced by X-ray signal from the inclusion. Additionally, two wavelength dispersive X-ray spectroscopy measurements (WDS) were performed in inclusion-free garnet at ample distance from any rutile inclusion in order to quantify the Ti-concentration in R1 (68–134 ppm, mean = 82 ppm TiO_2_, detection limit c. 40 ppm).

### Shape preferred orientations (SPOs)

**Fig. 4:**
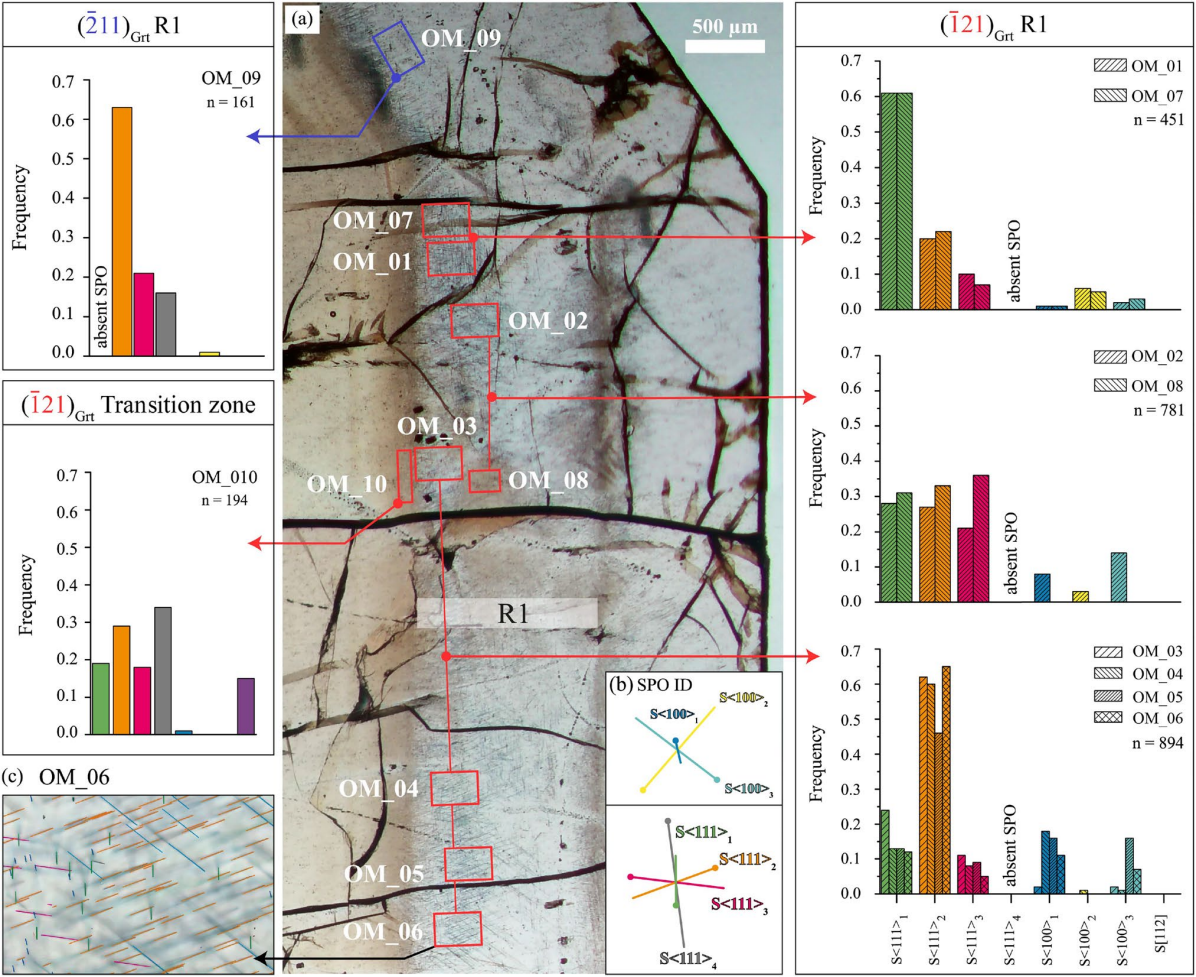
**a** Location of selected sample areas wherefrom the relative frequencies of rutile SPOs have been quantified. Areas OM-01 to -07 represent colourless garnet domains of R1 in the ($\overline{1}$21)_Grt_ sector. OM-08 represents a locally coloured garnet patch in otherwise colourless R1 of the same sector, whereas OM-10 represents the transition zone. Area OM-09 is located in R1 of the ($\overline{2}$11)_Grt_ sector. Results of the quantification of individual SPOs are displayed as bar charts (n = number of rutile needles counted in each area). OM domains with similar SPO frequencies are grouped. **b** Coloured lines show individual SPO directions (SPO ID) parallel to $\langle$111$\rangle$_Grt_ and $\langle$100$\rangle$_Grt_ with relative line lengths as projected onto the thin section plane and as observed with rutile needles in OM (compare with **c** and Fig. 1). The direction of needle plunge is indicated by the dot, which corresponds to the intersection with the thin section surface. **c** Exemplary image of the SPO quantification (area OM_06, $200 \times 300 \upmu$m). The complete documentation of all sample domains in which the SPO quantification was performed, is provided in the supplementary Fig. 11

For each studied rutile inclusion in garnet the elongation direction of the needle was obtained as projected onto the section plane, while qualitatively determining the direction and angle of plunge with respect to this plane. Based on these results, each rutile crystal could be assigned to one of a total of eight SPOs (Fig. 4b and Appendix Fig. 10). Correlating the elongation direction of rutile with the particular low indexed garnet direction to which the needle is parallel, yields three shape orientation relationships (SORs) between the two phases, with rutile SPOs parallel to $\langle 111 \rangle$, $\langle 100 \rangle$ and $\langle 112 \rangle$ directions of garnet (Figs. 1d, 4b and Appendix Fig. 10). A unique identification (SPO ID, Fig. 4) was assigned to each SPO, first by determining which particular garnet direction it was parallel to, then by numbering individual crystallographic equivalent directions. The number of rutile needles with a particular elongation direction was quantified in representative OM areas (OM-XX). The resulting SPO frequencies are given as fraction of the total amount of needles analysed for SPO in each OM area (Fig. 4). Subsequently, the SPO frequencies were examined for any correlation with the normal direction to the corresponding garnet facet, which is considered to represent the propagation direction of that facet during garnet growth (section ‘Rutile SPO and garnet growth direction’). Additionally, the effect of sample sectioning has been considered (section ‘Sectioning effect on SPO frequencies’).

#### Observed SPO frequencies and rutile-garnet SOR

The observed frequencies of the particular shape preferred orientations (SPOs) of rutile inclusions in the transition zone and R1 have been quantified (Fig. 4, Appendix Table 4 and Fig. 11). The ($\overline{1}$21)_Grt_ facet was studied in OM-01–OM-07 (R1 zone), OM-08 (shaded area in R1 zone) and OM-10 (transition zone). For comparison, the crystallographically equivalent growth facet ($\overline{2}$11)_Grt_, has been investigated in the representative area OM-09 (R1 zone). Considering the entire SPO dataset (transition zone and R1 zone), from a total of 2481 rutile inclusions in both studied garnet sectors, a total of eight SPOs of rutile were identified (Fig. 4 and Appendix Fig. 10). When these SPOs are correlated with the crystallographic orientation of garnet as determined by electron backscatter diffraction analysis (EBSD), the following SORs of the two phases are derived: four SPOs are parallel to the three-fold $\langle 111 \rangle$_Grt_ axes (in the following termed S$\langle 111 \rangle$_1_, S$\langle 111 \rangle$_2_, S$\langle 111 \rangle$_3_ and S$\langle 111 \rangle$_4_), three are parallel to the four-fold $\langle 100 \rangle$_Grt_ axes (termed S$\langle 100 \rangle$_1_, S$\langle 100 \rangle$_2_ and S$\langle 100 \rangle$_3_) and one is parallel to [112]_Grt_ (Fig. 4 and Appendix Fig. 10).

In the R1 zone of garnet, wherefrom the largest SPO dataset has been collected, six different SPOs are observed in each of the two garnet sectors that are associated with the ($\overline{1}$21)_Grt_ and ($\overline{2}$11)_Grt_ facets (noted with red and blue dashed line in Fig. 1a). There, rutile needle elongation directions parallel to $\langle 111 \rangle$_Grt_ prevail, whereas SPOs parallel to $\langle 100 \rangle$_Grt_ are present with lower abundance.

In the ($\overline{1}$21)_Grt_ sector, rutile needles in colourless and shaded domains of R1 (OM-01–OM-08) predominantly pertain to S$\langle 111 \rangle$_1_ or S$\langle 111 \rangle$_2_, whereas S$\langle 111 \rangle$_3_ is generally less frequent, and only one single rutile inclusion with SPO S$\langle 111 \rangle$_4_ was observed. S$\langle 111 \rangle$_2_ prevails in OM-03, -04, -05 and -06, whereas S$\langle 111 \rangle$_1_ is the most prominent needle orientation in OM-01 and OM-07, which are the OM areas closest to the adjacent ($\overline{2}$11)_Grt_ growth sector. In OM-02 the S$\langle 111 \rangle$_1_ and S$\langle 111 \rangle$_2_ are almost equal in abundance (n = 43 and 41, respectively), while the S$\langle 111 \rangle$_3_ is less abundant (n = 33). In all these cases rutile needles with S$\langle 111 \rangle$_1_ and S$\langle 111 \rangle$_2_ are more abundant than $\langle 111 \rangle$_3_ needles (Fig. 4). Colourless garnet domains of R1 show significantly lower abundance of rutile inclusions and consequently a larger inclusion spacing than in a shaded domain (OM-08, table 4). There, needle-shaped inclusions almost exclusively have elongation directions parallel to $\langle 111 \rangle$_Grt_ and S$\langle 111 \rangle$_2_ is more frequent than S$\langle 111 \rangle$_1_ and S$\langle 111 \rangle$_3_.

For comparison with the R1 zone of the intensely studied ($\overline{1}$21)_Grt_ sector, an area of R1 in a different, crystallographically equivalent sector corresponding to the ($\overline{2}$11)_Grt_ facet (OM-09, Fig. 4) has been investigated. Here, rutile needles with S$\langle 111 \rangle$_2_ also have the highest abundance. A striking difference between the two sectors is the absence of rutile inclusions with SPO S$\langle 111 \rangle$_1_ in the R1 zone of the ($\overline{2}$11)_Grt_ sector, whereas needles of S$\langle 111 \rangle$_4_ are much more abundant than in the ($\overline{1}$21)_Grt_ sector, where only one single inclusion following S$\langle 111 \rangle$_4_ was observed.

Contrasting with the SPO systematics of the R1 zone, rutile needles in the transition zone of the ($\overline{1}$21)_Grt_ sector show SPOs parallel to all four $\langle 111 \rangle$_Grt_ directions at rather equal abundances (OM-10). Rutile needles with S$\langle 111 \rangle$_4_ and S$\langle 111 \rangle$_2_ are only slightly more frequent than those with S$\langle 111 \rangle$_1_ or S$\langle 111 \rangle$_3_. Whereas rutile SPOs parallel to the $\langle 100 \rangle$_Grt_ are nearly absent in the transition zone (1 out of n = 194), a significant fraction of rutile crystals with SPO S[112] have been identified (Appendix Fig. 10). Interestingly, no rutile inclusion with an SPO parallel to any other crystallographically equivalent $\langle 112 \rangle$_Grt_ direction was observed.

Whereas SPO frequencies were quantitatively derived from the R1 zone and the transition zone of garnet, the inclusions in the outer garnet core only yielded qualitative SPO information due to their submicrometer size. Still, some results were obtained from scanning transmission electron microscopy (STEM) images of a FIB prepared foil of the ($\overline{1}$21)_Grt_ core sector (Appendix Fig. 9). In this domain, rutile crystals appear equant in OM, but show some non-equidimensionality in STEM images collected in viewing direction parallel to [$\overline{1}\overline{1}1$]_Grt_, corresponding to S$\langle 111 \rangle$_4_. Submicrometer sized rutile inclusions show elongation parallel to $[\overline{1}11]$_Grt_ or $[1\overline{1}1]$_Grt_, which correspond to S$\langle 111 \rangle$_2_ and S$\langle 111 \rangle$_3_, respectively. For inclusions of S$\langle 111 \rangle$_4_ the extent of elongation is unknown, as it is perpendicular to the foil plane.

Rutile needles elongated parallel to $\langle 100 \rangle$_Grt_ are almost exclusively restricted to the R1 zone of garnet. As their total numbers are too low for statistical relevance (n = 16 to 45 in OM-01–OM-07, supplementary Table 4), their relative frequencies have not been analysed. Further analysis and interpretation of SPO frequencies is, therefore, focused on rutile needles with elongation parallel to any of the $\langle 111 \rangle$_Grt_.

#### Rutile SPO and garnet growth direction

**Fig. 5:**
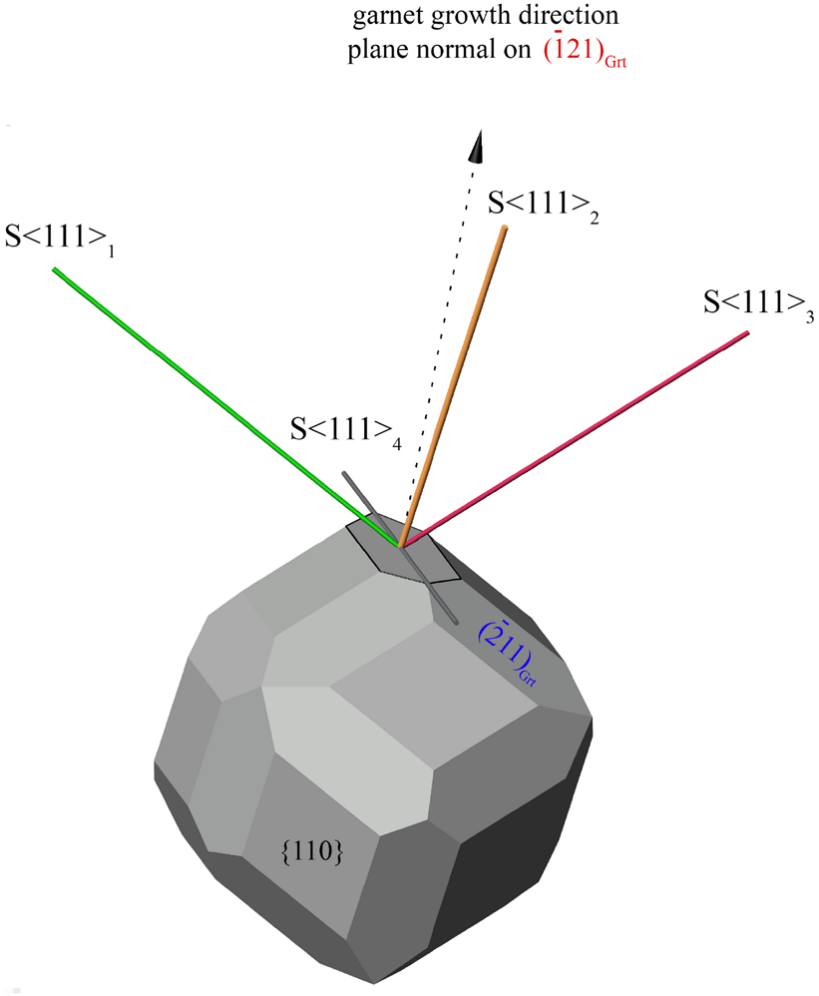
3D model of garnet with dodecahedral {110}_Grt_ and trapezohedral {112}_Grt_ facets illustrating the orientation of SPOs of rutile parallel to $\langle 111 \rangle$_Grt_ directions with respect to the ($\overline{1}$21)_Grt_ (marked facet) and the plane normal (black dashed arrow). The latter is supposed to correspond to the growth direction of this garnet sector, where SPO S$\langle 111 \rangle$_4_ lies in the facet plane (90^∘^ to the plane normal of the growth facet), while S$\langle 111 \rangle$_2_ has the smallest angle of 19^∘^ to the plane normal of the growth facet. SPOs S$\langle 111 \rangle$_1_ and S$\langle 111 \rangle$_3_ have the same angle of 62^∘^ to the plane normal of this particular facet

**Fig. 6:**
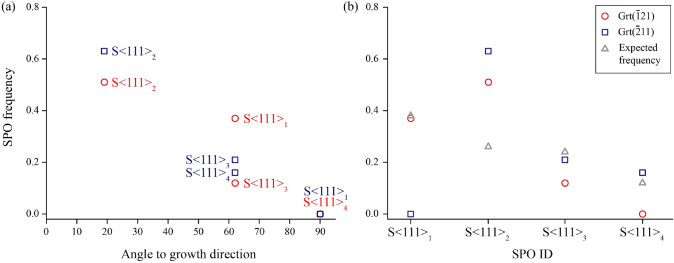
Plot of fractions of rutile needles with SPOs parallel to $\langle 111 \rangle$_Grt_ in the colourless R1 zone of garnet. **a** Observed relative SPO frequencies vs. angle between needle elongation and growth direction of garnet in the ($\overline{1}$21)_Grt_ sector (red circles) and the ($\overline{2}$11)_Grt_ sector (blue squares). The SPO with the smallest angle to the corresponding growth direction of garnet shows the highest frequency, whereas the SPO that is parallel to the growth facet of garnet is almost entirely absent (n = 1 in OM-07). **b** Estimated relative SPO frequencies considering only the sectioning effect (grey triangles), and observed relative frequencies of rutile needles with SPO parallel to any of the $\langle 111 \rangle$_Grt_ SPO for the ($\overline{1}$21)_Grt_ sector and the ($\overline{2}$11)_Grt_ sector

A potential correlation between the relative frequencies of rutile SPOs and the particular garnet growth direction (assumed to be perpendicular to the local garnet growth facet plane) has been investigated for rutile needles with SPO parallel to any of the $\langle 111 \rangle$_Grt_. The 3D model of the garnet crystal morphology (Fig. 5) illustrates the growth direction of the ($\overline{1}$21)_Grt_ facet (arrow with dashed line) and the elongation directions of the SPOs parallel $\langle 111 \rangle$_Grt_. The S$\langle 111 \rangle$_2_ direction has the lowest angle (19^∘^) with respect to the growth direction of garnet for both the ($\overline{1}$21)_Grt_ and ($\overline{2}$11)_Grt_ facet. For each facet, one particular SPO lies in the growth facet plane: S$\langle 111 \rangle$_4_ is parallel to the ($\overline{1}$21)_Grt_ facet, and S$\langle 111 \rangle$_1_ is parallel to the ($\overline{2}$11)_Grt_ facet (Table 1). For each garnet sector, two SPOs have the same angle to the garnet growth direction, namely S$\langle 111 \rangle$_1_ and S$\langle 111 \rangle$_3_ in the ($\overline{1}$21)_Grt_ sector, and S$\langle 111 \rangle$_3_ and S$\langle 111 \rangle$_4_ in the ($\overline{2}$11)_Grt_ sector. Plotting the SPO frequencies versus the angle of the rutile needle elongation with respect to the growth direction of garnet, we observe that for both sectors the most abundant SPO (S$\langle 111 \rangle$_2_) has the smallest angle with respect to the garnet growth direction. The particular SPO that is parallel to the facet plane is strikingly underrepresented or absent in each growth sector (Fig. 6a).

#### Sectioning effect on SPO frequencies

The cutting plane through the garnet crystal leads to different apparent spacing of the needle-shaped inclusions due to their different inclinations with respect to the section plane. When assuming equal spacing of the rutile needles orthogonal to their elongation direction, a potential bias by sample sectioning can be estimated (details in section ‘Shape preferred orientation (SPO) count’). Variations in relative frequencies of SPOs that are exclusively induced by the sectioning effect (*estimated* SPO frequencies) are independent of the growth facet orientation. Considering only rutile needles with SPO parallel to any $\langle 111 \rangle$_Grt_, the highest estimated frequency is determined for S$\langle 111 \rangle$_1_ (fraction 0.38). Similar inclination angles of rutile needles with S$\langle 111 \rangle$_2_ and S$\langle 111 \rangle$_3_ with respect to the section plane, yield comparable frequencies (fraction 0.26 and 0.24). The lowest estimated frequency is inferred for S$\langle 111 \rangle$_4_ (fraction 0.12) due to the shallow inclination (15^∘^) with respect to the section plane (Table 1).

**Table 1:** Angles of the rutile needle SPOs with respect to the plane normals of two particular garnet growth facets (each presumed to correspond to the growth direction of the corresponding garnet sector), and the total number and relative frequencies of rutile needles in the R1 zone

SPO ID	Rt elongation direction	$(\overline{1}21)_{Grt}$			$(\overline{2}11)_{Grt}$		
		Angle to Grt growth direction	Observed Rt needles	Observed frequency	Angle to Grt growth direction	Observed Rt needles	Observed frequency
S$\langle 111 \rangle _{1}$	$[111]_{Grt}$	62	483	0.37	90	0	0.00
S$\langle 111 \rangle _{2}$	[$\overline{1}11]_{Grt}$	19	668	0.51	19	101	0.63
S$\langle 111 \rangle _{3}$	$[1\overline{1}1]_{Grt}$	62	153	0.12	62	34	0.21
S$\langle 111 \rangle _{4}$	[$\overline{1}\overline{1}1]_{Grt}$	90	1	0.00	62	25	0.16
			1305			160	

For the Grt$(\overline{1}21)$ sector the areas OM-01 to OM-07 are representative for colourless garnet of the R1 zone, whereas for the Grt$(\overline{2}11)$ sector OM-09 serves for comparison

Figure 6b provides a comparison of *observed* versus *estimated* relative SPO frequencies at equal spacing orthogonal to needle elongation. It illustrates that the *observed* relative SPO frequencies greatly differ from those that can be referred to the sectioning effect. Rutile needles with S$\langle 111 \rangle$_2_ are much more frequent in both garnet sectors than could be explained by the sectioning effect. The particular SPO which is parallel to the growth facet plane of garnet, is absent or extremely rare (n = 1, Table 1), although needles with S$\langle 111 \rangle$_1_ (absent in the ($\overline{2}$11)_Grt_ sector) are supposed to have the highest frequency at equal spacing orthogonal to needle elongation. Also, 12% (156 rutile needles) of the total number of 1305 rutile needles elongated parallel to any of the $\langle 111 \rangle$_Grt_ in OM-01 to OM-07 should pertain to S$\langle 111 \rangle$_4_ when equal spacing orthogonal to the needle elongation is assumed. Contrastingly, only a single inclusion of S$\langle 111 \rangle$_4_ was actually observed in the colourless domains of R1 in the ($\overline{1}$21)_Grt_ sector.

### Crystallographic orientation relationships (CORs)

Crystal orientations of a total of 354 rutile inclusions with different habit were collected by EBSD single-spot analyses in two areas covering the three microstructural domains of the ($\overline{1}$21)_Grt_ sector, the outer core (n = 38, exclusively equant rutile in OM), the transition zone (n = 40, exclusively rod-shaped rutile) and the R1 zone (n = 276, exclusively needle-shaped rutile). The garnet host is a single crystal and thus has the same orientation in all microstructural domains.

The categorisation of the EBSD dataset is performed with a Matlab script for the MTEX toolbox, modified after Griffiths et al. (2020). Details of the procedure are given in section ‘Electron backscatter diffraction (EBSD) analysis’ and in the supplementary section “Description of categorisation method”. The orientations of rutile inclusions with respect to the garnet host are categorised by pairs of parallel crystal directions in garnet and rutile (Griffiths et al. 2020), and labeled according to Hwang et al. (2016). In addition, a significant amount (n = 18) of all rutile inclusions in the new EBSD dataset showed a previously unreported COR, which is defined by the parallel directions or plane poles $\langle 103\rangle _{\text{Rt}}\Vert \langle 111\rangle _{\text{Grt}}$, $\langle 100 \rangle$_Rt_$\Vert \{112\}$_Grt_ and $\langle 320 \rangle$_Rt_$\Vert \{120\}$_Grt_ (note that $\langle$uvw$\rangle \Vert${hkl} in this case refers to *one* of the symmetrically equivalent rutile $\langle$uvw$\rangle$ directions being parallel to *one* of the symmetrically equivalent garnet {hkl} plane poles). On the basis of the notation by Hwang et al. (2015, 2016), where COR labels are assigned according to the end-on orientation of rutile needles in TEM, the corresponding $\langle 111\rangle$_Grt_ or $\langle 001\rangle$_Grt_ zone axis, and a certain orientation derived from rotation around this axis, the newly identified COR is denoted as COR-3^#^. It is related to COR-3 of Hwang et al. (2016) by a c. 10^∘^ rotation of rutile about $\langle 103\rangle _{Rt}\Vert \langle 111\rangle _{Grt}$. Further definition of this COR is provided in the supplementary section “Definition of COR-3^#^”.

**Fig. 7:**
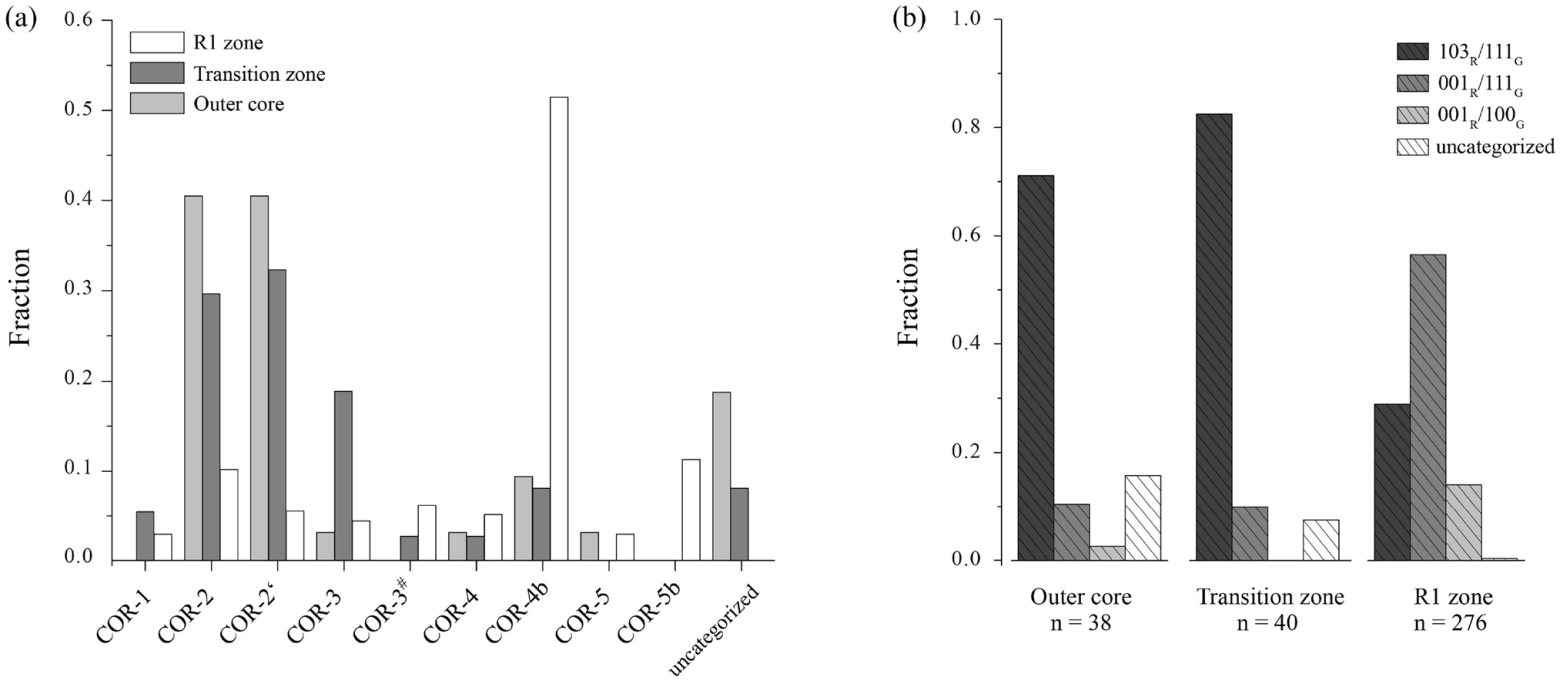
**a** Fraction of specific CORs for each microstructural domain based on the entire COR dataset (n = 354). CORs tested for but not found are omitted from the x-axis. Only inclusions with no confirmed COR are counted in “uncategorised”. **b** Fraction of rutile inclusions pertaining to particular COR groups for each microstructural domain

Considering the entire EBSD dataset (n = 354), which covers all three microstructural domains of garnet and different rutile habits (equant, rod- and needle-shaped), the crystallographic orientation relationships COR-4b, COR-2, COR-2’ and COR-5 are the most common (Fig. 7a and Table 2). In the outer core the prevalent CORs are COR-2 and COR-2’. Six inclusions were uncategorised, two of which were also assigned to uncategorised^+ax^. The transition zone is dominated by COR-2’ and COR-2, followed by COR-3. Three inclusions are uncategorised, with one assigned to uncategorised^+ax^. In contrast to the outer core and the transition zone, the most abundant COR in R1 zone is COR-4b, followed by COR-5 and then COR-2. Only one inclusion is uncategorised. In summary, we find similarities between the outer core and transition zone, which are both dominated by COR-2 and COR-2’ and have a relatively high fraction of uncategorised inclusions. In contrast, the R1 zone is dominated by COR-4b and COR-5, which are both minor in the aforementioned domains and all except for one inclusion could be categorised to one of the known CORs (Fig. 7a).

#### COR groups

Crystallographic orientation relationships (CORs) of rutile inclusions and garnet host crystals show much larger variability than SPOs. Eleven different non-equivalent CORs have been previously identified for rutile inclusions in garnet host crystals (Hwang et al. 2016; Griffiths et al. 2016, 2020), with one more conclusively identified in the new dataset. Griffiths et al. (2020) emphasised the potential of using relative frequencies of CORs in this complex system to infer petrogenetic information from particular samples and garnet domains. In order to evaluate the relative frequencies of identified CORs in the new EBSD dataset, we apply the approach of pooling CORs based on common axial relationships between rutile inclusions and the garnet host crystal. The resulting COR groups are defined by particular parallel (/) crystal directions in garnet (G) and rutile (R). The following three COR groups comprise 97.2% of the rutile inclusions in the studied dataset (they also cover 100% of the 344 inclusions with a specific COR in this dataset, as well as nine of the 12 rutile-garnet CORs hitherto known):

- Group 103_R_/111_G_ is defined by one of the $\langle 103 \rangle$_Rt_ parallel to one of the four $\langle 111 \rangle$_Grt_. Rutile inclusions of the CORs that are part of this group have their c-axis arranged along a cone around any of the $\langle 111 \rangle$_Grt_ (Proyer et al. 2013; Hwang et al. 2015, 2016; Griffiths et al. 2016). This COR group pools several specific CORs, namely COR-1, COR-2, COR-2’, COR-3 and COR-3^#^, as well as four inclusions that follow this axial relationship but no known specific COR (uncategorised^+ax^).
- Group 001_R_/111_G_ is defined by $\langle 001 \rangle$_Rt_ parallel to one of the four $\langle 111 \rangle$_Grt_ and comprises COR-4 and COR-4b.
- Group 001_R_/100_G_ is defined by $\langle 001 \rangle$_Rt_ parallel to one of the three $\langle 100 \rangle$_Grt_ and comprises the specific CORs termed COR-5 and COR-5b.

**Table 2:** Number of rutile inclusions pertaining to specific CORs, and pooled into COR groups, given for the entire dataset (n = 354), and listed separately for three particular microstructural zones of garnet

Specific CORs and COR groups	Outer core	Transition zone	R1 zone	Total
**103**_R_/**111**_G_	**27**	**33**	**80**	**140**
COR-1	0	2	8	10
COR-2	13	11	28	52
COR-2’	13	12	15	40
COR-3	1	7	12	20
COR-3^#^	0	1	17	18
uncategorised^+ax^	2	1	1	4
**001**_R_/**111**_G_	**4**	**4**	**156**	**164**
COR-4	1	1	14	16
COR-4b	3	3	142	148
**001**_R_/**100**_G_	**1**	**0**	**39**	**40**
COR-5b	1	0	8	9
COR-5	0	0	31	31
**Sum COR groups**	**32**	**37**	**275**	**344**
uncategorised	6	3	1	10
% categorised	84.2%	92.5%	99.6%	97.2%
sum all	38	40	276	354

“Uncategorised” comprises all inclusions that do not attain any of the 12 known specific CORs, some of which share the axial relationship of CORs in Group 103_R_/111_G_ (uncategorised^+ax^)

#### Correlation of SPOs and CORs in the garnet R1 zone

**Fig. 8:**
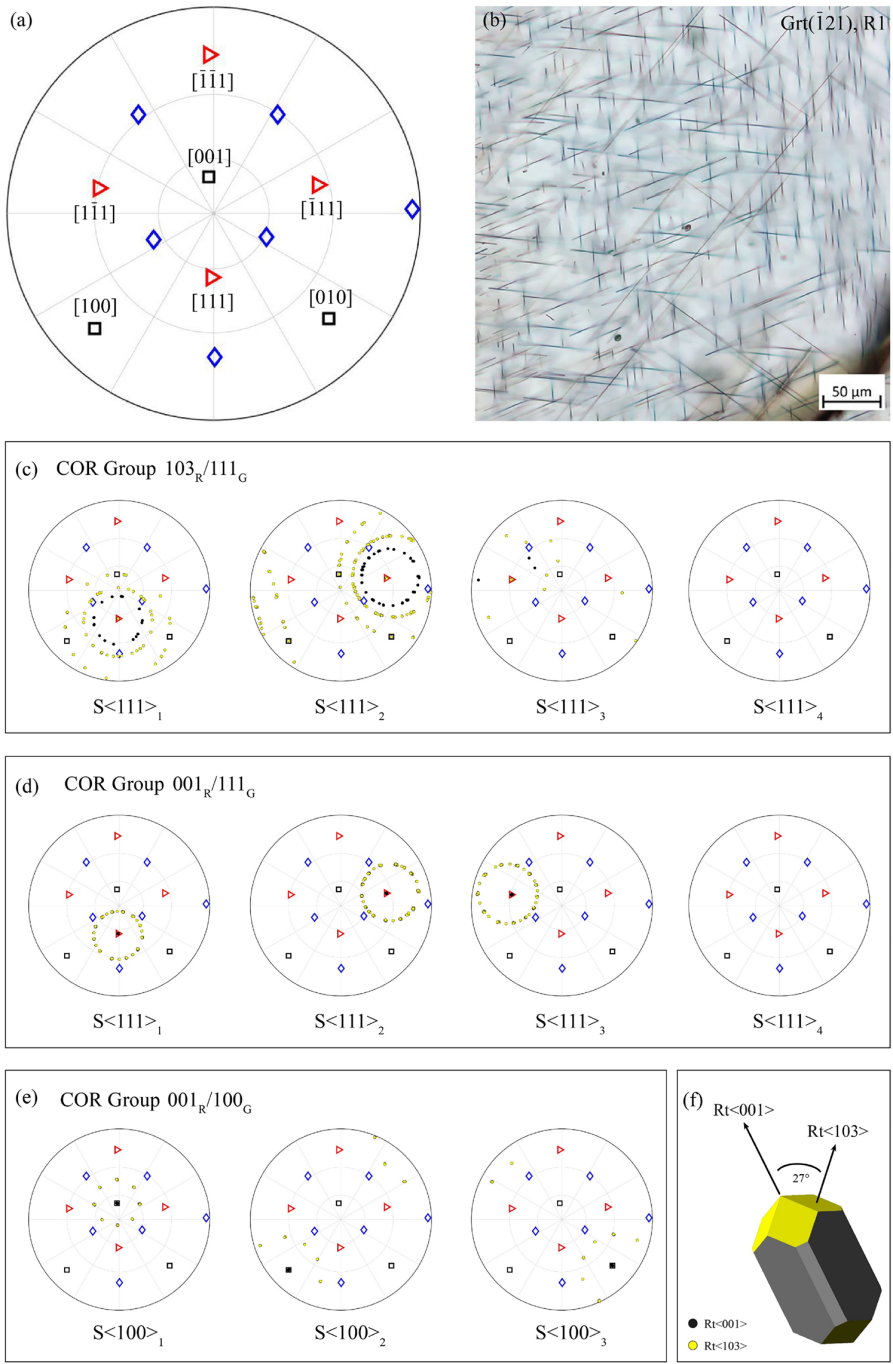
SPO-COR correlation of needle-shaped rutile inclusions in the R1 zone of the ($\overline{1}$21)_Grt_ sector. The same reference frame is used for EBSD crystal orientation data (pole figures as upper hemisphere equal angle projection) and the photomicrograph (OM-TLp). **a** Pole figure showing the garnet crystal directions $\langle 111\rangle$_Grt_ (red triangles), $\langle 100\rangle$_Grt_ (black squares), $\langle 110\rangle$_Grt_ (blue diamonds). **b** OM-TLp image shows needle-shaped rutile inclusions with six SPOs. **c**–**e** EBSD crystal orientation data of needle-shaped rutile inclusions and garnet host filtered by COR group and SPO. The axial relationship defining the COR group coincides with the particular elongation direction of rutile needles: **c** Rutile inclusions of COR Group 103_R_/111_G_ only show needle elongation S$\langle 111 \rangle$_1_, S$\langle 111 \rangle$_2_, or S$\langle 111 \rangle$_3_. **d** Rutile inclusions of COR Group 001_R_/111_G_ exclusively show needle elongation S$\langle 111 \rangle$_1_, S$\langle 111 \rangle$_2_, and S$\langle 111 \rangle$_3_. **e** Rutile inclusions of COR Group 001_R_/100_G_ exclusively have SPOs S$\langle 100 \rangle$_1_, S$\langle 100 \rangle$_2_ or S$\langle 100 \rangle$_3_. **f** Morphological model of rutile showing the four {405}_Rt_ planes in yellow and the c-axis

The EBSD crystal orientation dataset for garnet host and rutile inclusions was combined with information on the SPO and the microstructural domain of each rutile inclusion measured. Filtering the EBSD dataset obtained from needle-shaped rutile in the R1 zone of the ($\overline{1}$21)_Grt_ sector by the COR group and a particular SPO reveals a systematic correlation between the shape and crystallographic orientation relationships (Fig. 8). Either the [001]_Rt_ or one of the $\langle 103 \rangle$_Rt_ is always parallel to the morphological elongation of the rutile needles. Furthermore, the morphological elongation direction of rutile needles is always parallel to the particular rutile direction that is aligned with a low-indexed garnet direction. Inclusions elongated parallel to [001]_Rt_ pertain either to the COR Group 001_R_/100_G_ or to the COR Group 001_R_/111_G_. On the other hand, rutile inclusions with elongation parallel to $\langle 103 \rangle$_Rt_ exclusively correspond to the COR Group 103_R_/111_G_. Thus, rutile needles with one of the three SPOs parallel to $\langle 100 \rangle$_Grt_ exclusively represent COR Group 001_R_/100_G_, whereas rutile needles with elongation parallel to $\langle 111 \rangle$_Grt_ either belong to COR Group 103_R_/111_G_ or to COR Group 001_R_/111_G_.

#### Relative COR group frequencies in different microstructural domains

In the R1 zone of garnet 99.6% of the measured rutile inclusions are part of one of the three COR groups defined in section ’COR groups’. This is the case for 92.5 and 84.2% of the rutile inclusions measured in the transition zone, and the outer core, respectively. For the entire EBSD dataset (not discriminating by rutile habit), rutile inclusions with an axial relationship involving the $\langle 111 \rangle$_Grt_ (Group 103_R_/111_G_ and Group 001_R_/111_G_) are the most abundant.

The relative frequencies of inclusions belonging to the three COR groups were quantified based on all EBSD crystal orientation data in each of the three microstructural domains of garnet (Fig. 7b). The equant rutile from the outer core and rod-shaped rutile from the transition zone show similarities in the frequencies of the COR groups. In both zones, group 103_R_/111_G_ CORs are prevalent (> 70%), whereas Group 001_R_/111_G_ is minor (11% and 10% in the outer core and transition zone, respectively). Group 001_R_/100_G_ is very rare (3% n = 1) in the outer core, and absent in the transition zone. Contrastingly, we detect a distinct change in the relative frequencies of the COR groups from the transition zone to the R1 zone: for the needle-shaped inclusions in R1, Group 001_R_/111_G_ is predominant (57%), and there is a remarkably higher abundance of Group 001_R_/100_G_ (14%). Accordingly, the frequency of Group 103_R_/111_G_ CORs is significantly lower (29%) in R1 compared to the other microstructural zones.

## Discussion

### Facet-specific SPO frequencies of rutile needles in garnet

Quantitative shape preferred orientation (SPO) data from a statistically relevant number of rutile needles in the garnet rim zone R1 show systematic differences in the fractions of particular rutile SPOs when comparing data from R1 in two different crystallographically equivalent growth sectors (Fig. 4). The quantification of SPO frequencies is based on the SPOs parallel to $\langle 111 \rangle$_Grt_, whereas the SPOs parallel to $\langle 100 \rangle$_Grt_, which are present in lower quantity, are discussed only qualitatively (Appendix Table 4). The consistent SPO data in domains far from a sector boundary (OM-03, -04, -05 and -06, $(\overline{1}21)$_Grt_, Fig. 4) are regarded as most representative for the studied sector and were used for the comparison with the SPO frequencies of rutile inclusions in the $(\overline{2}11)$_Grt_ sector. This comparison yielded striking results: SPO S$\langle 111\rangle$_4_ is absent in the $(\overline{1}21)$_Grt_ sector, whereas it is present in a significant amount in the corresponding growth zone of the $(\overline{2}11)$_Grt_ sector (OM-09, Fig. 4). On the other hand, SPO S$\langle 111\rangle$_1_ is absent in $(\overline{2}11)$_Grt_ sector, but present in a considerable quantity in the $(\overline{1}21)$_Grt_ sector. We can exclude that these variations in SPO frequencies are due to an analytical bias or an effect of the section orientation, as clearly illustrated by SPOs S$\langle 111 \rangle$_2_ and S$\langle 111 \rangle$_3_, which both have a similar inclination with respect to the thin section plane (Fig. 4b and methods section). These two SPOs should be observed at equal fraction if the differences in SPO frequencies were exclusively induced by a sectioning effect. Their absolute amounts differ, however, by a factor of 6.6 in the representative domains. Furthermore, based on the total amount of 776 needles in the representative R1 domains of the $(\overline{1}21)$_Grt_ sector, we would expect 93 needles in SPO S$\langle 111 \rangle$_4_ orientation if the sectioning effect was the only control on the SPO frequency. Instead, S$\langle 111 \rangle$_4_ is entirely absent in these domains (Fig. 6 and Table 1).

Finally, in both studied sectors, the SPO with the lowest angle (19^∘^) to the garnet growth direction is much more abundant than expected based on the sectioning effect (Table 1). Therefore, the local growth direction of garnet has a selective effect on the SPO frequencies in the studied sample.

### Origin of oriented needle-shaped rutile inclusions in garnet

Conspicuous differences in the SPO and COR characteristics of rutile needles in three microstructural domains of garnet along the transition from core to R1 and in two garnet growth sectors (Figs. 4 and 7), impose constraints on the temporal and spatial relationship of garnet and rutile crystallisation in the studied sample. An earlier study on this material by Kohn et al. (2024) has shown that the concentric zones of the outer core, the transition zone and the R1 zone of garnet have formed in a single magmatic growth stage, despite the striking microstructural differences (Fig. 1c).

According to Griffiths et al. (2020), SPO and COR frequency data are supposed to allow discrimination between a solid-state exsolution origin of needle-shaped inclusions in a pre-existing host crystal, and a co-growth origin of the host and inclusion phases. Especially, the comparison of two crystallographically equivalent growth sectors of the host crystal is considered to yield conclusive information. Furthermore, rather high crystal symmetry of those sectors allow for several equivalent host crystal directions, which potentially control the SPO of inclusion phases. The new correlated SPO-COR dataset of rutile inclusions in a garnet host crystal provides a statistically representative set of quantitative SPO frequency data that are directly linked with the COR data, and also allow the sectioning effect on SPO frequencies to be accounted for. Following the approach of Griffiths et al. (2020), we discuss three hypothetical relative time relationships between the crystallisation of the garnet host and the rutile inclusions in the light of the new SPO frequency dataset, and evaluate arguments that support or rebut each scenario. We consider (i) overgrowth of pre-existing rutile by garnet, (ii) intragranular solid state precipitation in pre-existing garnet and (iii) simultaneous growth of rutile and garnet.

In the concept of (i) overgrowth, rutile nucleates and grows before being engulfed by garnet to form inclusions. Euhedral rutile is usually elongated parallel to the c-axis, which would result in straight elongation-parallel extinction in the optical microscope using crossed polarised light. Instead, a significant fraction of rutile inclusions in all microstructural zones of garnet show oblique extinction. In the R1 zone of garnet, these inclusions all pertain to the COR Group 103_R_/111_G_, which is characterised by rutile needle elongation parallel to one of the $\langle 103 \rangle$_Rt_ (Fig. 7 and Table 2). Due to this uncommon habit, another solid phase is inferred to impose the morphology on rutile. As needle elongation exclusively follows the low indexed $\langle 111 \rangle$_Grt_, $\langle 100 \rangle$_Grt_ and one of the $\langle 112 \rangle$_Grt_ directions (Fig. 4b, Appendix Fig. 10), the presence of garnet during rutile formation seems to be a requirement.

Hypothetically, the SPO of rutile needles could also trace another crystalline precursor phase that had been topotactically replaced by garnet, like sagenitic biotite (Shau et al. 1991) or hornblende (Mongkoltip and Ashworth 1983). However, the SPO and crystallographic orientations of rutile needles would then be expected to reflect the symmetry of the precursor phase rather than that of garnet. Based on the clearly documented SPOs and CORs between rutile needles and garnet in the studied sample, an overgrowth scenario can be excluded.

In the case of (ii) intragranular solid-state precipitation of rutile within pre-existing garnet, we expect equal frequencies of crystallographically equivalent COR variants, and equal frequencies of SPOs parallel to crystallographically equivalent garnet directions to form in a homogeneous host crystal (Boudeulle 1994; Griffiths et al. 2020). Potentially, rutile nucleation on growth defects could induce unequal equivalent SPO and/or COR frequencies via precipitation. However, such a mechanism is supposed to result in linear microstructural features normal to the growth front of garnet (Hwang et al. 2015). Due to the lack of chains, “tubes” or inclusions aligned subparallel to the assumed growth direction of the studied garnet, we can exclude a significant effect of growth dislocations on the observed SPO- and COR-frequencies. Also, an effect of externally imposed strain on the defect content of the host crystal, and thus on the SPO/COR frequency and/or the inclusion distribution is considered irrelevant to crystallisation in a magmatic system.

Based on the above, the absence of the particular SPO that is parallel to the corresponding facet plane of garnet in R1 of each of the two studied sectors (Figs. 5 and 4), and the presence of only one of twelve $\langle 112\rangle$_Grt_ SPO variants in the transition zone (Appendix Figs. 10, 11), clearly contradicts the hypothesis of precipitation within a pre-existing garnet crystal for the origin of rutile inclusions in these garnet domains.

Another feature that could pinpoint an inclusion origin by exsolution from a Ti-component in garnet is the presence of a depletion halo of Ti adjacent to rutile needles (Ague and Eckert 2012; Axler and Ague 2015a). A previous investigation of the studied sample has shown, that the pervasive presence of nanoinclusions in the garnet core impedes the analysis of the minor and trace element composition in this garnet domain (Kohn et al. 2024). Thus, the section of the Ti profile covering the garnet core comprises a contribution by rutile nanoinclusions (Fig. 2b) rendering the detection of any depletion halos in this garnet domain impossible. Contrastingly, a detailed Ti-profile in garnet adjacent to rutile needles in the R1 zone documents the absence of Ti zoning (Fig. 3). Furthermore, there are no inclusions of silicate phases associated with rutile needles in the R1 zone of garnet, rendering unlikely that rutile formed by exsolution of a Ti-component from garnet in a closed intragranular chemical system (Proyer et al. 2013). In case that diffusion had led to Ti depletion of garnet in R1 zone, or enabled material exchange with the rock matrix to form rutile by open system precipitation (Proyer et al. 2013), we would expect the Na profile in garnet to have rehomogenised. Instead, the preserved Na zoning in garnet (Fig. 3) clearly opposes a scenario of intragranular precipitation of rutile. Therefore, consistent with the systematic differences in SPO frequencies in equivalent garnet growth sectors (Fig. 6), the compositional data also contradict an exsolution scenario for this sample.

Finally, the hypothesis of (iii) co-growth of garnet host and rutile inclusions is compatible with the observation of differing microstructural characteristics of inclusion phases in different crystallographically equivalent sectors, correlating with the local growth direction of garnet. The selective effect of the particular garnet facet on the SPO frequencies (Fig. 6), as verified in this study, is consistent with qualitative observations in metapegmatite garnets from Koralpe (Eastern Alps, Austria, Griffiths et al. 2020). These authors recognised shorter or absent $\langle 111 \rangle$_Grt_ SPOs of rutile needles oriented parallel to $\{112\}$_Grt_ growth facets, whereas longer and more abundant ones are oriented with a high angle to these growth facets. Griffiths et al. (2020) regarded this observation as indicative of inclusion formation by “oriented interface nucleation and subsequent simultaneous crystallisation”. In this mechanism, rutile is proposed to nucleate on a pre-existing garnet facet, with the COR determined at the moment of nucleation. The correlation of SPOs and CORs for needle-shaped rutile inclusions in this study (Fig. 8) implies that the potential SPO is likely also determined at the moment of nucleation. As the observed SPO and COR frequencies of rutile inclusions clearly depend on the local propagation direction of their associated garnet growth facet, the co-growth hypothesis is the most likely time relationship for rutile needles in garnet for the transition zone and growth zone R1 of the studied sample. Several recent observations that support scenarios of simultaneous formation of rutile inclusions and host crystallisation considering garnet (Hwang et al. 2015; Griffiths et al. 2020) or corundum host crystals (Palke and Breeding 2017), indicate, that this timing relationship is becoming increasingly relevant, and should additionally be regarded, besides the established precipitation model.

### Correlation of SPOs and COR groups of rutile-needles in garnet

There is a strict correlation between the SPOs of needle-shaped rutile inclusions in zone R1 and the proposed COR groups (Fig. 8). The morphological elongation of the needle-shaped rutile crystals is parallel to the common pair of parallel crystal directions in garnet and rutile, which define the corresponding COR group. For the studied sample, 99.6% of the needles in the R1 zone of garnet are categorised as one of these three COR groups (Table 2). Here, variations in observed SPO frequencies are thus directly correlated with variations in the frequencies of these COR groups.

A comparison of the new dataset with available SPO-COR data in the literature shows consistent correspondences of the shape preferred orientations of rutile needles and the proposed COR groups, although SPO and COR data are often not directly linked, and/or the sizes of the datasets are very limited. A consistent correlation of SPO $\langle 111 \rangle$_Grt_ and CORs belonging to COR Group 103_R_/111_G_ was reported by Hwang et al. (2015). Proyer et al. (2013) report $\langle 111 \rangle$_Grt_ SPOs which are predominantly associated with CORs assigned to COR Group 103_R_/111_G_, and only subordinately with COR Group 001_R_/111_G_. These two COR groups also represent the predominant orientation relationships between rutile needles and garnet host in a growth zone toward the rim of a garnet in metapegmatite from the Wirtbartl locality, Koralpe, Austria (Griffiths et al. 2020). In addition to the orientation relationships described above, though always at lower frequency, needle-shaped rutile inclusions with $\langle 100 \rangle$_Grt_ SPOs and belonging to COR Group 001_R_/100_G_, were reported from 6-star garnet (Hwang et al. 2015).

Based on this correspondence of the SPOs and CORs, we recommend to pool CORs into COR groups (Table 2) based on the common axial relationships when comparing statistically representative SPO/COR frequency data from different sample domains, such as different garnet generations, growth zones or sectors within a particular sample. It is crucial to note that conclusive comparison of COR-datasets derived from EBSD measurements on different sample material is only possible if the same categorisation procedure is applied to all datasets. Provided that the crystal orientation of the host garnet is known, the most common three COR groups of needle-shaped rutile inclusions can already be distinguished by optical microscopy based on their SPO and extinction angle: Rutile needles with SPO $\langle 100\rangle$_Grt_ exclusively represent COR Group 001_R_/100_G_. Rutile needles with SPO $\langle 111\rangle$_Grt_ and straight extinction represent COR Group 001_R_/111_G_, whereas needles with SPO $\langle 111\rangle$_Grt_ and oblique extinction represent COR Group 103_R_/111_G_.

Due to the large variability of potential specific CORs between rutile and garnet, their relative frequencies are considered to bear additional petrogenetic information (Griffiths et al. 2020, with references). However, in order to reliably quantify the relative frequencies of specific CORs, very large datasets of several hundreds of rutile inclusion orientations are required from each homogeneous garnet domain. Further, COR frequencies from needle-shaped rutile are biased by the sectioning effect, as needles at the highest angle with respect to the sample surface will constitute the largest fraction, while needles subparallel to the sample surface can be missed. Also, given the elongated shape of the interaction volume for EBSD measurements, the needle orientation with respect to the beam orientation during EBSD analysis may cause some bias due to selective analysis when needle cross sections have widths of significantly less than c. 500 nm. Therefore, provided that needle-shaped rutile overwhelmingly pertain to the three main COR groups described above, the statistical evaluation of SOR and COR group frequencies based on optical light microscopy and EBSD analysis of garnet represents a favourable approach to rapidly yet convincingly constrain the relative time relationship between rutile inclusion and garnet host crystallisation. However, as the grouping is based on the correlation of SPO and COR data for needle-shaped habit, it is not applicable to equant inclusions or those which lack information on their elongation direction.

### Comparison of different garnet growth zones in the same growth sector

When comparing different growth zones of the $(\overline{1}21)$_Grt_ growth sector, characteristic changes in the morphology, crystal size, SPO and COR characteristics of rutile inclusions were observed (Figs. 1c, 4 and 7). The garnet core and the transition zone show similarities regarding the colour of garnet, the presence of rutile with all four $\langle 111 \rangle$_Grt_ SPOs, and a prevalence of COR group 103_R_/111_G_ with dominant COR-2 and COR-2’ (Fig. 4, Table 2). Due to the similar characteristics of the garnet core and the transition zone, both domains are henceforth discussed together as “core domains”. Likely, the co-growth formation mechanism, as inferred from the SPO observations for the transition zone and R1 (section ‘Origin of oriented needle-shaped rutile inclusions in garnet’), also applies to the outer core. Nonetheless, a separate origin for inclusions in the outer core cannot be completely ruled out, as statistical SPO data are not available for this domain.

By contrast with the “core domains”, the R1 zone is characterized by colourless garnet, rutile needles with up to 150 micrometer length, and the absence of rutile parallel to the particular growth facet of the corresponding garnet sector (Fig. 6). In the R1 zone, rutile needles with SPO $\langle 111 \rangle$_Grt_ predominantly pertain to CORs of Group 001_R_/111_G_. Additionally, needles with SPO $\langle 100 \rangle$_Grt_ and the associated COR Group 001_R_/100_G_ constitute a significant fraction of rutile inclusions. The identified differences in the microstructural and textural features when comparing the rutile inclusions in the core domains and R1 must reflect changes in parameters during progressive growth of the garnet host. As the garnet core, the transition zone and the growth zone R1 formed in a single magmatic growth stage, changes in PT conditions are supposed to be of minor relevance (Kohn et al. 2024). Instead, the physico-chemical properties of the bulk melt and of a compositional boundary layer (CBL) at the garnet-melt interface need to be considered. Especially the viscosities of the bulk melt and the CBL influence the rates of material transport, and thus the local melt composition and its supersaturation with respect to rutile and garnet. Accordingly, changes in the chemical driving force for the nucleation and growth of garnet and rutile may result.

A previous study showed that the R1 zone of garnet has slightly higher OH^–^ content than the garnet core, while Na increases and Ti decreases across the transition zone (Kohn et al. 2024). These authors related the change in the garnet composition to an increase in the H_2_O-content and in the availability of SiO_2_ in the pegmatoid melt, corresponding to an increase in the anorthite component of coexisting plagioclase. Also, they describe quartz-inclusions present in the R1 zone, but absent in the garnet core and the rock matrix (Kohn et al. 2024). This implies, that the garnet core crystallised at SiO_2_-undersaturated conditions, whereas the availability of SiO_2_ increased at the transition to the formation of the R1 zone. Therefore, changes in melt composition, either in the bulk melt or in a compositional boundary layer at the garnet-melt interface, have to be considered when trying to understand the cause for the conspicuous microstructural differences between the core domains and growth zone R1.

Furthermore, Kohn et al. (2024) reported effects of sector-specific local compositional boundary layers (CBLs) on microstructure formation during crystallisation of the garnet core. In general, fractional crystallisation of pegmatoid melt systems increase the concentration of elements which are incompatible in the primary magmatic mineral assemblage. The presence of fluxing elements (such as fluorine, boron, phosphorous) as well as H_2_O increases diffusion rates in the melt and leads to rather low nucleation rates with respect to crystal growth rates. The latter defined as the propagation rate of the local crystal-melt interface in the direction normal to the growth facet plane. These melt characteristics result in the formation of the particularly coarse-grained magmatic assemblages. Especially, relatively high growth rates of the host may promote the local saturation of second phases at the growth front (London 1992; Simmons and Webber 2008), and thus, potentially lead to a local supersaturation with respect to phases to which the bulk melt is still undersaturated. This is reflected by the absence of rutile in the rock matrix, whereas rutile inclusions are ubiquitously present in garnet (Kohn et al. 2024). Contrasting with the stage of garnet core crystallization, during the formation of R1 the increased H_2_O content of the melt is expected to decrease the viscosity of the melt and increase bulk diffusion rates of components in the melt. The consequent widening of the CBL and lowering of the supersaturation with respect to garnet and rutile at the solid-melt interface is hypothesised to decrease the chemical driving force for rutile nucleation and to potentially change the relative growth rates of rutile and garnet (Kohn et al. 2024).

When comparing the microstructures of the transition zone and R1, relative differences in the abundance and the aspect ratio of rutile inclusions are evident (Fig. 1c). At least qualitatively, these microstructural characteristics can be used to infer changes in (i) the relative rates of rutile nucleation and growth, as well as (ii) the relative growth rates of garnet and rutile during co-growth. Firstly, while the transition zone is characterised by a high abundance of relatively short, rod-shaped rutile inclusions, the R1 zone is characterised by a lower overall abundance of rutile inclusions, which show significantly higher aspect ratios. This indicates that the ratio of rutile nucleation rate to rutile growth rate was higher during the formation of the transition zone than during formation of R1. Secondly, when the local growth rate of garnet exceeds the growth rate of a given rutile inclusion measured parallel to the same direction, that rutile inclusion will be engulfed and stop growing. Given the lower aspect ratio of inclusions in the transition zone, we infer that the probability of engulfment, and thus the ratio of the garnet growth rate to the rutile growth rate, was relatively higher in the transition zone compared to R1. The effect of the changes in the melt properties reported by Kohn et al. (2024), are thus in agreement with the observed changes in the microstructures defined by rutile inclusions. We conclude that the lower supersaturation of components at the garnet-melt interface during growth of the R1 zone had an observable effect on the rate of nucleation and growth of rutile and is responsible for the strong change in habit and abundance of rutile inclusions.

Still, the radial variations in frequency of different SPO-COR combinations, and the change in microstructure of R1 within a single sector cannot be conclusively explained by changes in melt composition and relative growth rates. Accordingly, in the following, we discuss further parameters that may have changed with time to provoke the observed radial variations (section ‘Radial variations in SPO and COR frequencies’), and subsequently, parameters that potentially varied spatially along the interface during the formation of R1 (section ‘SPO variations within growth zone R1’).

### Radial variations in SPO and COR frequencies

Rutile is absent in the rock matrix, whereas the garnet core hosts numerous rutile inclusions that are inferred to have initially formed at the garnet-melt interface (Kohn et al. 2024). Thus, heterogeneous nucleation of rutile at the garnet-melt interface seems to be energetically favoured with respect to nucleation in the melt.

Importantly, the parameters that determine which SPO-COR combinations *nucleate* and which ones *grow* to form visible inclusions, may be different. The evidence of a selective effect of the local garnet growth direction on the SPO statistics in the rim (section ‘Facet-specific SPO frequencies of rutile needles in garnet’), suggests that inconvenient rutile needle orientations either do not grow to a significant size, or that such SPO-COR combinations do not nucleate at all. A selective effect on the nucleation is dominated by the interface energy, as SPO-COR combinations that minimise the 2D interface energy of rutile and garnet are more favourable to form. As the interface between garnet and rutile increases upon co-growth, a selection of SPO-COR combinations that allow for low-energy interfaces (minimisation of the strain and interface energy) in 3D is possible (Keller and Ague 2022). The fact that the outer core and the transition zone have very similar COR characteristics (Fig. 7), although the aspect ratio significantly differs in both domains (Fig. 1c), leads to the conclusion that the selective effect of co-growth after nucleation was not as important for the development of COR characteristics in this period of inclusion habit change, while a selective effect during nucleation seems to be more relevant. Still, the drastic change of SPO-COR characteristics between the core domains and R1 remains to be clarified.

Regarding heterogeneous nucleation on an existing garnet surface, it is obvious that the atomic configuration of that facet is highly relevant for the COR formation. Accordingly, the variable configurations of macroscopic {112}_Grt_ facets on the atomic and sub-micron scales need to be highlighted. Macroscopic {112}_Grt_ facets may exhibit different microtopographies on sub-micron scale, as they can be composed of stepped {110}_Grt_ facets (Kretz 2010; Gulbin and Glazov 2013) at atomic scale. Also, {112}_Grt_ facets can be decorated with growth spirals and stripes elongated parallel to particular $\langle$111$\rangle$_Grt_ directions resulting in interface steps with heights in the range of a few angstöm (Milke 2004). At the atomic level, two different configurations of {112}_Grt_ facets may theoretically exist, depending on how the garnet unit cell is cut, resulting in specific cations of either Mg and Al, or Si and Al exposed at the garnet/melt interface (Boutz and Woensdregt 1993). Furthermore, the atomic configuration of the garnet facet changes when crossing the roughening temperature transition during growth (Sunagawa 2007), which additionally changes the growth mechanism that establishes the advancement of the garnet growth front.

While we cannot correlate particular atomic configurations of the garnet facet to particular SPO and COR trends at present, we have to account for considerable variations in the nature of the garnet facets on submicron to atomic-scale. The exclusive appearance of $\langle 100\rangle$_Grt_ SPOs in R1 (Fig. 4) may point to a potential change in the atomic configuration between the {112}_Grt_ core domains and the rim, and accordingly to a change in the growth mechanism of garnet. We conclude that the conspicuous changes in the SPO frequencies and corresponding CORs (Fig. 7) may be related to changes in the atomic configuration of the garnet facet, as these would directly affect the SPO-COR combinations and variants that are energetically favoured to form.

### SPO variations within growth zone R1

Variations within a growth zone are unlikely directly related to petrological variables, but rather provide insight into details of inclusion formation at a particular time step. As the microtopography of a garnet facet is not necessarily homogeneous (Lefever and Chase 1962; Gulbin and Glazov 2013), local lateral variations have to be taken into account. Although the atomic configuration and microtopography of the {112}_Grt_ facet at the time of rutile nucleation are not directly observable, the divergent SPO frequencies in selected domains may give some indications. The similar SPO frequencies of S$\langle 111\rangle$_1–4_ in OM-02 and OM-08, when compared to the representative domains OM-03, -04, -05 and -06 in the laterally central domains of the facet, may be influenced by local characteristics of the garnet/melt interface and the growth mechanism of the advancing growth front (Fig. 4). Also, the different garnet colour in the shaded area of R1 (OM-08) may point to deviating conditions of inclusion formation. Furthermore, the location adjacent to the sector boundary (OM-01 and -07) shows significantly different SPO frequencies when compared to the representative domains. Domains located close to a sector boundary may be influenced by lateral concentration gradients (Berg 1938), or laterally differing growth mechanisms associated with the formation of re-entrant zones (Rice and Mitchell 1991), possibly affecting the local SPO- and COR-characteristics. These observations indicate locally differing conditions for rutile nucleation along a single facet, and highlight the importance of careful domain selection when SPO and/or COR data are acquired.

### Summary and comparison with another pegmatite

The pronounced change in the microstructural characteristics from garnet core to R1 growth observed with the studied sample is interpreted to reflect changes in the relative rates of growth and nucleation of rutile, as well as the relative rates of garnet and rutile growth. These are referred to changes in the composition (Na, Si and OH^–^) and viscosity of the melt at the onset of the growth of the transition zone, and a decreased supersaturation of the melt with respect to rutile at overall high growth rates of all phases during pegmatoid solidification (Kohn et al. 2024). Finally, the atomic configuration of the garnet facet may have determined the energetically favoured CORs for nucleation. For needle-shaped rutile, the geometrical relationship of the garnet growth direction and the needle orientation determines which particular SPOs are more likely to form.

A similar microstructural development has been reported from garnet in metapegmatite from the Koralpe, locality Wirtbartl, in the Eastern Alps (Griffiths et al. 2020). These authors describe a coloured garnet core hosting equant rutile inclusions along with corundum and phosphates, in contrast to a colourless garnet rim that bears kyanite and needle-shaped rutile inclusions (the latter showing facet-related absences of particular SPO variants) and is intergrown with quartz. The obvious SiO_2_-deficiency in the garnet core, and the increased availability of SiO_2_ during formation of the garnet rim, are similar to the studied sample. So are the microstructural and SPO characteristics as well as some COR features of rutile and garnet. In both cases, the garnet core domains that formed under SiO_2_ deficient conditions comprise predominantly equant rutile with considerable or prevailing COR Group 103_R_/111_G_, whereas rutile needles in the colourless garnet growth zone associated with quartz predominantly pertain to the COR Group 001_R_/111_G_.

These systematic changes in magmatic garnet host-inclusion microstructures from similar lithologies from two different geological units, widely separated in time and space thus indicate similarities, not only in the process active during crystallisation, but also in the evolution of both systems which we attribute to similar trends in changing pegmatitic melt composition upon fractionation.

## Conclusions

A new EBSD dataset yields the crystallographic orientation relationships (CORs) between > 350 rutile inclusions and their garnet host crystal in a pegmatoid, keeping track of the microstructural domain, the crystal habit of each inclusion measured and the shape preferred orientation (SPO) of each inclusion with high aspect ratio. The stand-alone SPO dataset from > 2400 rutile needles quantifies the frequency of individual needle elongation directions in a garnet rim zone of two crystallographically equivalent Grt{112} growth sectors. Results from the correlated investigation of microstructures, crystallographic textures and garnet compositional zoning lead to the following conclusions:

- Needle and rod-shaped rutile inclusions show clear shape orientation relationships with the garnet host. Eight distinguishable shape preferred orientations of rutile needles reflect three shape orientation relationships with the garnet host crystal. Rutile needles are elongated along the four Grt$\langle 111\rangle$, the three Grt$\langle 100\rangle$ directions and one particular Grt$\langle 112\rangle$ direction.
- SPOs and CORs of needle-shaped rutile inclusions and the garnet host are clearly correlated. Based on these correspondences, we propose pooling specific CORs into COR groups when comparing datasets from different host crystal domains or different samples. The proposed COR groups 103_R_/111_G_, 001_R_/111_G_, 001_R_/100_G_ can be identified by polarised light microscopy if the garnet crystal orientation is known.
- The local growth direction of garnet has a selective effect on SPO frequencies: the most abundant rutile needles are those with an SPO closest to the garnet growth direction, whereas rutile inclusions with an SPO parallel to the garnet growth facet are absent.
- The control of SPO frequencies of rod- and needle-shaped rutile inclusions by the particular garnet growth direction in each sector is consistent with inclusion formation by co-growth (Griffiths et al. 2020). The development of particular SPO-COR combinations supposedly are influenced by the atomic configuration of the garnet facet in the moment of nucleation.
- Changes in SOR- and COR-characteristics are referred to changes in the structural and compositional nature of the garnet/melt interface, melt composition and properties, and/or the growth mechanism of the garnet host. While garnet core domains formed under rather Si-deficient conditions, the rutile-needle bearing garnet rim is inferred to have crystallised from a melt with higher Na, Si and OH^–^ content. According changes in the melt viscosity are considered to induce a decrease in the nucleation rate of rutile with respect to its growth rate.
- Observed microstructural and compositional features of the studied pegmatoid garnet bearing numerous rutile inclusions are consistent with those reported from a metapegmatite from the Austroalpine Koralpe crystalline basement (Griffiths et al. 2016). Similarities of both samples involve the co-growth origin of rutile inclusions and a growth related effect on the SPO frequencies. Most importantly, changes in COR group frequencies associated with increasing Si- (and OH^–^) content of the melt seem to be a systematic feature of magmatic fractional crystallisation in peraluminous pegmatoids and pegmatites.

### Supplementary Information

Below is the link to the electronic supplementary material.

Supplementary File 1: S1 EPMA data (XLSX 12 KB)

Supplementary File 2: S2 EBSD data (XLSX 58 KB)

Supplementary File 3: S3 SPO data (XLSX 12 KB)

Supplementary File 4 (PDF 16070 KB)

Appendix Fig. 10z

## Data Availability

Information regarding the availability of the research data has been added to the “Acknowledgements” and the “Supplementary Material” section.
