# Supplementary material for: Variations in orientation relationships between rutile inclusions and garnet host relate to magmatic growth zoning
Source: Contrib Mineral Petrol. 2024 Jun 17;179(7):69. doi: 10.1007/s00410-024-02146-9 (PMC11182869; doi:10.1007/s00410-024-02146-9)
Supplement: Supplementary file 4 — (PDF 16070 KB) [file 410_2024_2146_MOESM4_ESM.pdf]

Appendix

Description of categorisation method

The categorisation method used in this paper is identical in principle to that employed by Griffiths et al. (2020). By means of an MTEX script, the rutile inclusions were filtered according to whether particular crystallographic directions or plane poles of rutile followed axial relationships with particular garnet directions, within a set angular threshold (each axial relationship plus threshold is referred to as a "rule" in this work, table 3). The filtering process can be visualised as a flowchart (as in Figure A1 of Griffiths et al., 2020). Rules are tested sequentially, and only the inclusions that fulfill the prerequisites for a given rule are tested according to that rule. Inclusions that follow the tested rule are selected to be filtered further or, if the rule is the final one required to define a COR, assigned to that COR. Inclusions that do not fulfill a tested rule may also be filtered further, assigned to a particular COR (this occurs if a filtered population of inclusions contains only inclusions belonging to two CORs, so that applying a rule sorts them into two different CORs), or assigned as "uncategorised" if no further rules are available and no COR was assigned according to the last rule checked. Because of the flowchart-like structure of the filtering process, it is never possible to assign a single inclusion to multiple CORs. This structure also allows the application of rules which, had they been applied to the whole dataset, would not correctly separate inclusions with different CORs. In other words, the order in which the rules are applied is important in some cases – certain inclusions must be filtered out before applying other rules. Figure A1 of Griffiths et al. (2020) presents a particular set of rules and a sequence of their application, which allows 11 different CORs to be distinguished. However, in fact, order only matters for some of the rules applied – therefore the flowchart in Griffiths et al. (2020) represents one of several possible sequences of operations that will give identical results. This issue is addressed in our new formulation of applied rules, introduced in table 3 and elaborated on below. It should be noted that the sequence of rules necessary to uniquely classify an inclusion as belonging to a specific host-inclusion COR does not necessarily correspond to a precise definition of that COR, which for a specific COR requires specifying three non-parallel inclusion directions and/or plane poles and the crystallographic directions in the host these correspond to. For the 11 specific rutile-garnet CORs previously reported in the literature, this information is available in Hwang et al. (2016), and we give it in the section 'Definition of COR-3#' below for the newly proposed COR-$3^{\#}$.

In the current work, the majority of rules applied were identical to those used in Griffiths et al. (2020). Most exceptions involve an increase in the threshold angle used for individual rules (table 3). This was done to avoid excluding inclusions with orientations very close to a known COR, given the fact that dispersional statistical CORs concentrated around particular axial relationships but with deviations of up to 5° are well known for rutile in garnet (Griffiths et al., 2016). The choice of threshold size was based on an overview of all previously published EBSD datasets (Griffiths et al., 2016; Keller and Ague, 2019; Griffiths et al., 2020) in combination with the new data collected – CORs observed to show greater dispersion in at least one dataset were assigned larger threshold angles. Thresholds were increased for 9 rules, by between

0.5 and 3.5°. Given that application of the new rules leads to negligible differences in COR frequency compared to the results of Griffiths et al. (2020) (see main methods section), we confirm that these thresholds are large enough to cover the variability of all studied datasets without including previously uncategorised inclusions. For two of the 11 CORs previously known, small modifications were made to the applied rules beyond changing angular thresholds. One of the rules eventually leading to identification of inclusions following COR 6 was changed, because the previous work used a rule involving a range of angles (from 2 to 5.5°). All other rules use a simple $>$ or $<$ and an angular threshold. Therefore, a new rule was implemented, using an axial relationship identified by Hwang et al. (2016). This had negligible effects on the results of the filtering algorithm, as already mentioned. Finally, the order of application of the two rules used to identify inclusions belonging to the R3b COR was swapped. The axial relationship $\langle 100\rangle_{\mathrm{Rt}} \parallel \langle 112\rangle_{\mathrm{Grt}}$ is common to many CORs, so it was decided to test for this rule later in the sequence, to ensure that a maximum of inclusions had been filtered out. Again, the effect on results was negligible.

One new rule was added in order to separate out inclusions belonging to the newly proposed COR, named COR-$3^{\#}$. It should be noted that this new rule is applied to inclusions that would be classed as uncategorised according to the Griffiths et al. (2020) flowchart. For this reason, its application does not affect the frequency of the 11 other CORs plotted in the previous paper. However, applying the new rule would slightly reduce the amount of uncategorised inclusions in some datasets compared to the previous algorithm. Concretely, for the EBSD datasets studied by Griffiths et al. (2020), filtering for the new COR finds two (frequency 1.9%) COR-$3^{\#}$ inclusions in “core domain A”, four (2.8%) COR-$3^{\#}$ inclusions in “core domain B”, one (7.1%) COR-$3^{\#}$ inclusion in the “04T26K_rim (needles)” dataset, and one (1.1%) COR-$3^{\#}$ inclusion in the Connecticut dataset.

Supplementary table 3 presents all of the rules used to categorise inclusions in this work, along with any differences to the previous set of rules used. The new presentation as a table allows a standard description of all rules, including the information about which rules must be carried out before others. Rules are organised into three hierarchy levels (denoted by N, N.N, and N.N.N, from highest to lowest, where N is an integer, as well as by different grey shading in table (table 3). The numbering of rules indicates the relationship between them. For example, rules 1.1 and 1.2 are applied only to inclusions that follow rule 1, and rules 1.1.1 and 1.1.2 are only applied to inclusions following both rule 1 and rule 1.1. Rules of a lower hierarchy level must therefore always be tested after the corresponding rule(s) of the higher hierarchy level(s). For rules of the same hierarchy level, the order in which rules are carried out is in most cases not important, because there is no overlap between the rules. Only certain rules *must* be carried out after other rules of the same hierarchy level, to prevent overlaps. These particular rules, of which there are only three, are specifically indicated in table (table 3).

Rule	Rutile direction / plane pole	Garnet direction	Threshold angle (°)	Specific COR	Rules that must be true for COR	Order restrictions of rules	Changes vs. Griffiths et al. 2020
1	<103>	111	<2	-	-	-	Unchanged
1.1	<100>	112	<2	-	-	-	Unchanged
1.1.1	{101}	113	<6	COR 3	1, 1.1 and 1.1.1	-	Unchanged
1.1.2	{101}	113	>6	COR-3#	1, 1.1 and 1.1.2	-	New COR
1.2	<100>	134	<3.5	-	-	after 1.1	Unchanged
1.2.1	<100>	112	<7	COR 2'	1, 1.2 and 1.2.1	-	Unchanged *
1.2.2	<100>	112	>7	COR 2	1, 1.2 and 1.2.2	-	Unchanged *
1.3	<100>	110	<3	COR 1	1 and 1.3	-	Unchanged
2	<001>	111	<5.5	-	-	-	Threshold +0.5°
2.1	<100>	112	<5.5	COR 4	2 and 2.1	-	Threshold +3.5°
2.2	<110>	110	<5.5	COR 4b	2 and 2.2	-	Threshold +0.5°
3	<001>	100	<5	-	-	-	Unchanged
3.1	<100>	100	<5	COR 5b	3 and 3.1	-	Threshold +3.5°
3.2	<110>	100	<5	COR 5	3 and 3.2	-	Threshold +3°
4	<001>	110	<5	-	-	-	Unchanged
4.1	<100>	112	<5	R1	4 and 4.1	-	Unchanged
5	<122>	100	<3	-	-	after 1 & 2	Different axial relationship
5.1	{101}	120	<3	-	-	-	Threshold +1°
5.1.1	<100>	112	<3	COR 6	5, 5.1 and 5.1.1	-	Threshold +1°
6	{101}	110	<4	-	-	after 1, 2 & 5	Swapped with rule 6.1, threshold +1°
6.1	<100>	112	<5	R3b	6 and 6.1	-	Swapped with rule 6, threshold +1°

Table 3 Definition of the combination of precise axial relationships of rutile and garnet (Rules, within a given threshold angle) that define a specific COR. The hierarchy of rules defines the order in which they need to be applied. * these rules are unchanged with regard to Griffiths et al. (2020). However, they are incorrectly written in figure A1 of that paper due to a typographical error. The rules as written here are correct for both papers

Definition of COR-3#

COR-3$^{\#}$ is a new COR belonging to the $\langle 103\rangle_{\mathrm{Rt}} \parallel \langle 111\rangle_{\mathrm{Grt}}$ group. As with all members of this group, one $\langle 103\rangle_{\mathrm{Rt}}$ direction is parallel to one $\langle 111\rangle_{\mathrm{Grt}}$, and one of the two rutile a-axes lies in the $\{111\}_{\mathrm{Grt}}$ plane that is perpendicular to the shared $\langle 103\rangle_{\mathrm{Rt}} \parallel \langle 111\rangle_{\mathrm{Grt}}$ axis pair. For COR-3$^{\#}$, the a-axis in this plane is parallel ($< 1°$ average deviation) to a $\langle 123\rangle_{\mathrm{Grt}}$ direction. This configuration allows the second rutile a-axis to be parallel ($< 1°$ average deviation) to one of the $\langle 112\rangle_{\mathrm{Grt}}$ directions lying on a different $\{111\}_{\mathrm{Grt}}$ plane from that containing the first a-axis. COR-3$^{\#}$ shares two axial relationships ($\langle 103\rangle_{\mathrm{Rt}} \parallel \langle 111\rangle_{\mathrm{Grt}}$ and $\langle 110\rangle_{\mathrm{Rt}} \parallel \langle 112\rangle_{\mathrm{Grt}}$) with COR-3, but these two CORs are crystallographically distinct, with the misorientation angle between the two being c. $< 10°$. If the shared $\langle 103\rangle_{\mathrm{Rt}} \parallel \langle 111\rangle_{\mathrm{Grt}}$ axis pair is indexed as $[111]_{\mathrm{Grt}} \parallel [103]_{\mathrm{Rt}}$, the misorientation characterising COR-3$^{\#}$ can be precisely obtained using the axial relationships $[321]_{\mathrm{Grt}} \parallel [0\bar{1}0]_{\mathrm{Rt}}$ and $[121]_{\mathrm{Grt}} \parallel [100]_{\mathrm{Rt}}$. For this ideal formulation, if the axial relationships for the a-axes are fixed at 0° deviation, $[111]_{\mathrm{Grt}}$ is inclined just 0.79° to $[103]_{\mathrm{Rt}}$. Such a small angular deviation is close to the precision of the EBSD data, so it is not possible to evaluate which of the three axial relationships actually has the lowest angular deviation in the studied sample.

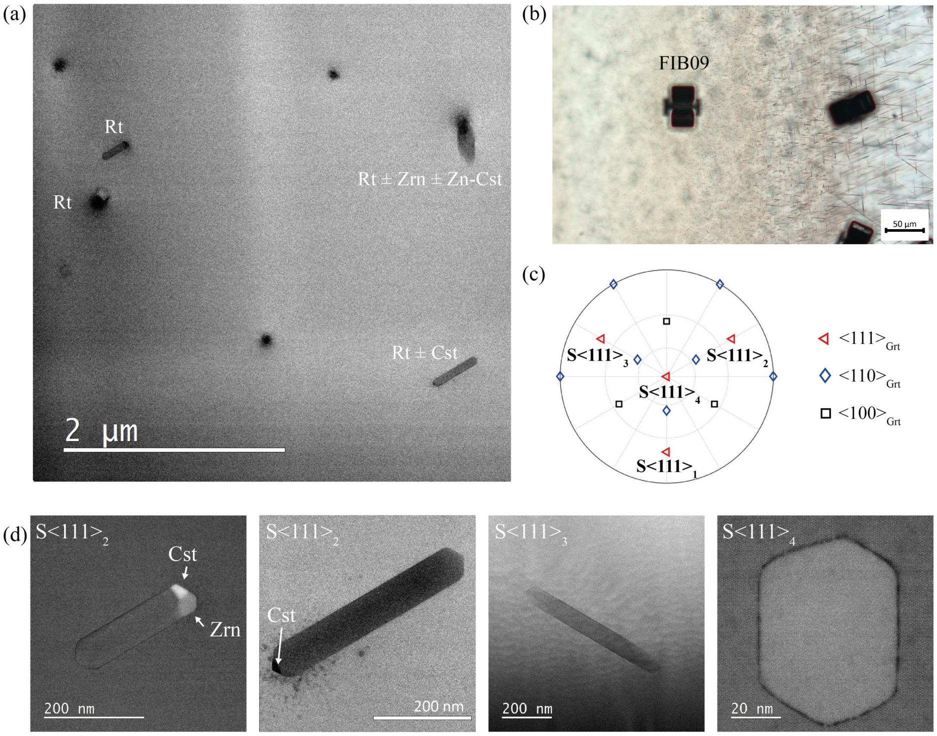

Fig. 9 (a) Overview of TEM foil FIB09 containing nanometer-sized rutile inclusions and polymineralic inclusions of rutile, cassiterite (Cst) and zircon (Zrn). TEM foil stems from the outer core, location given in Fig. 3 in Kohn et al. (2024). (b) Sample location in the outer core of the ($\bar{1}21$) sector. (c) Pole figure in foil view direction. (d) Detailed dark-field images (first, third and fourth image) and bright-field image (second image) of elongated Rt inclusions oriented along specific SPO directions. Regarding rutile elongated in foil view direction $S\langle 111\rangle_4$, the third dimension is not observable. However, the fact that the Rt-Grt interface is viewed edge-on, is consistent with an inclusion elongation along $S\langle 111\rangle_4$

SPO ID	Garnet direction	OM_01	OM_02	OM_03	OM_04	OM_05	OM_06	OM_07	OM_08	OM_09	OM_10
$\langle 111\rangle_1$	[111]	152	43	110	20	18	17	123	189	0	37
$\langle 111\rangle_2$	$[\bar{1}11]$	51	41	280	93	65	93	45	207	101	56
$\langle 111\rangle_3$	$[1\bar{1}1]$	26	33	48	12	13	7	14	222	34	35
$\langle 111\rangle_4$	$[\bar{1}\bar{1}1]$	0	0	0	0	0	0	1	0	25	65
$\langle 100\rangle_1$	[001]	2	12	7	28	23	16	3	0	0	1
$\langle 100\rangle_2$	[100]	14	4	2	1	0	0	10	1	1	0
$\langle 100\rangle_3$	[010]	4	21	7	1	22	10	6	0	0	0
[112]	[112]	0	0	0	0	0	0	0	0	0	29
all <111>		229	117	438	125	96	117	183	618	160	193
all <100>		20	37	16	30	45	26	19	1	1	1
n		249	154	454	155	141	143	202	619	161	194
all $\langle 111\rangle$		92%	76%	96%	81%	68%	82%	91%	100%	99%	99%
all $\langle 100\rangle$		8%	24%	4%	19%	32%	18%	9%	0%	1%	1%
$\langle 111\rangle_1$	[111]	0.61	0.28	0.24	0.13	0.13	0.12	0.61	0.31	0.00	0.19
$\langle 111\rangle_2$	$[\bar{1}11]$	0.20	0.27	0.62	0.60	0.46	0.65	0.22	0.33	0.63	0.29
$\langle 111\rangle_3$	$[1\bar{1}1]$	0.10	0.21	0.11	0.08	0.09	0.05	0.07	0.36	0.21	0.18
$\langle 111\rangle_4$	$[\bar{1}\bar{1}1]$	0.00	0.00	0.00	0.00	0.00	0.00	0.00	0.00	0.16	0.34
$\langle 100\rangle_1$	[001]	0.01	0.08	0.02	0.18	0.16	0.11	0.01	0.00	0.00	0.01
$\langle 100\rangle_2$	[100]	0.06	0.03	0.00	0.01	0.00	0.00	0.05	0.00	0.01	0.00
$\langle 100\rangle_3$	[010]	0.02	0.14	0.02	0.01	0.16	0.07	0.03	0.00	0.00	0.00
[112]	[112]	0.00	0.00	0.00	0.00	0.00	0.00	0.00	0.00	0.00	0.15

Table 4 Results of the SPO quantification in numbers of needles observed and the according frequency of each SPO from the total number of needles observed in each OM area

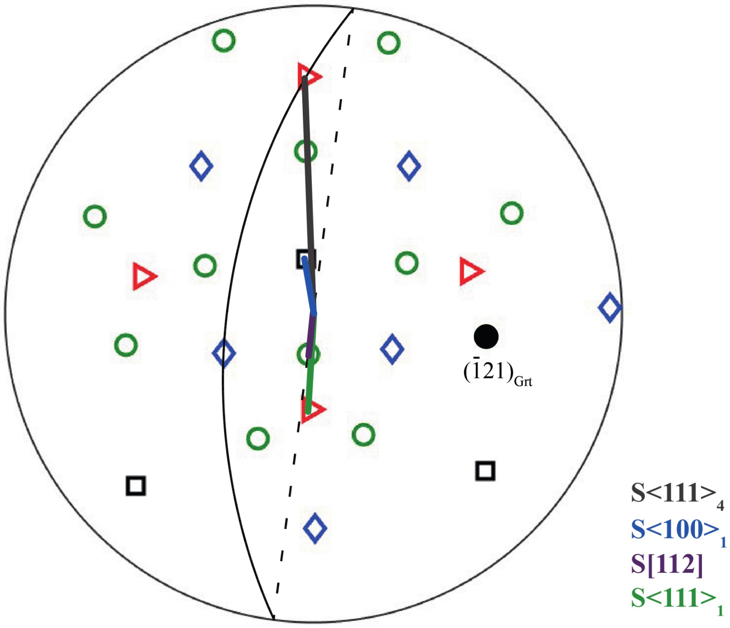

Fig. 10 Upper hemisphere pole figure showing the garnet orientation and the trace particular SPOs of oriented needle-shaped rutile inclusions: S$\langle 100\rangle_1$ in blue, S$\langle 111\rangle_1$ in green, S$\langle 111\rangle_4$ in gray and S[112] in purple. Great circle shows the $(\bar{1}21)_{\mathrm{Grt}}$ facet and the trace of the plane as dashed line

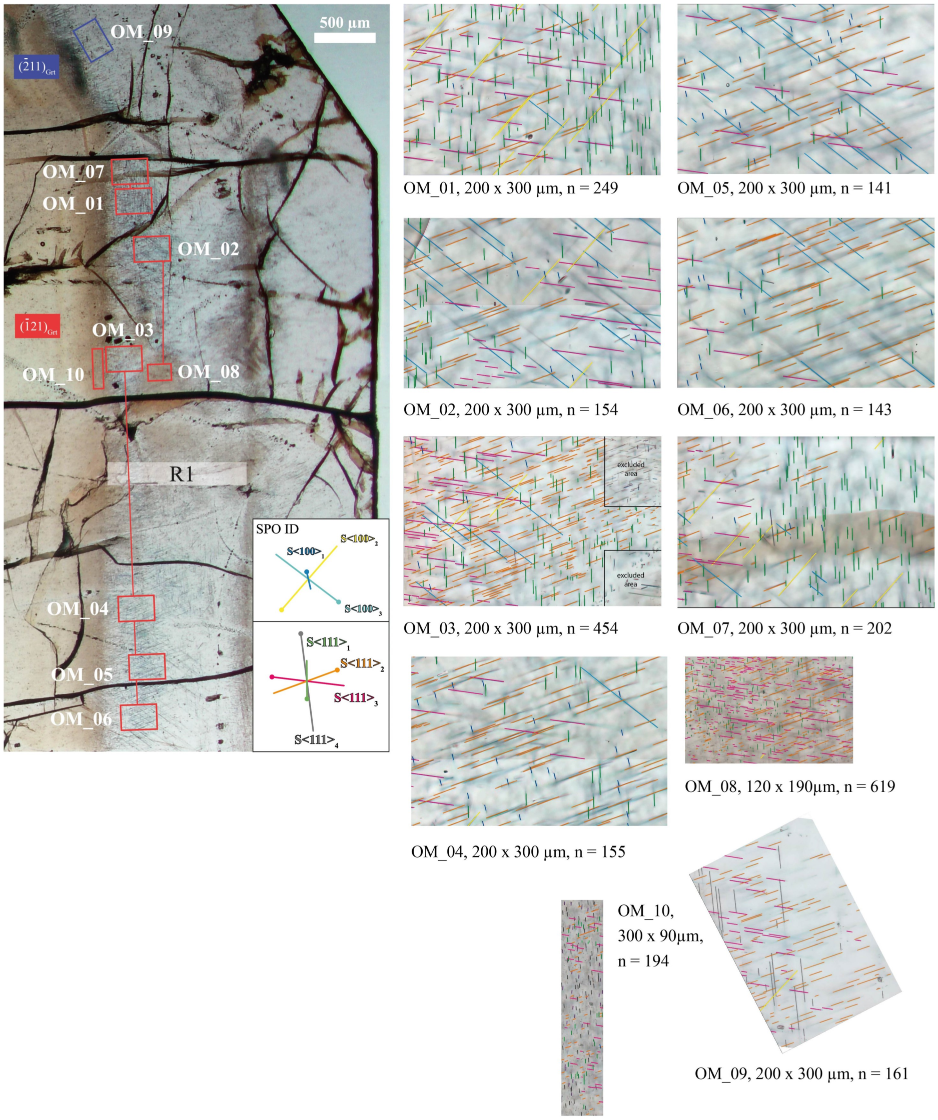

Fig. 11 Documentation of the SPO quantification. SPO 8 (purple) is visualised in figure 10.

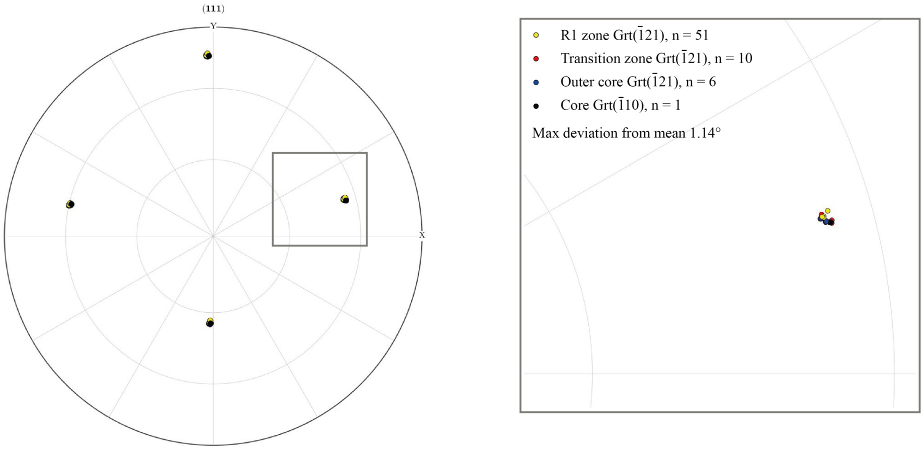

Fig. 12 Upper hemisphere pole figure of garnet ⟨111⟩ orientations combined for the domains R1, Transition zone, $\{112\}_{\mathrm{Grt}}$ core, $\{110\}_{\mathrm{Grt}}$ core. Grid lines = 30°. The mean angle of all measurements was determined. The maximum angle for all the garnet measurements from their mean is 1.14°. The studied garnet domains represent a single crystal.
